# PTRF-IL33-ZBP1 signaling mediating macrophage necroptosis contributes to HDM-induced airway inflammation

**DOI:** 10.1038/s41419-023-05971-1

**Published:** 2023-07-15

**Authors:** Juan Du, Yahui Liu, Gelei Lan, Yao Zhou, Yingmeng Ni, Kai Liao, Fang Zheng, Qijian Cheng, Guochao Shi, Xiao Su

**Affiliations:** 1 Department of Pulmonary and Critical Care Medicine, Ruijin Hospital, Shanghai Jiao Tong University School of Medicine, Shanghai, China; 2 Shanghai Key Laboratory of Emergency Prevention, Diagnosis and Treatment of Respiratory Infectious Diseases, Shanghai, China; 3 Department of Pediatrics, Ruijin Hospital, Shanghai Jiao Tong University School of Medicine, Shanghai, China; 4 Laboratory of Cell Biology, Institute of Biochemistry and Cell Biology, Shanghai Institutes for Biological Sciences, Chinese Academy of Sciences, Shanghai, China; 5 Department of Immunology, School of Basic Medicine, Tongji Medical College, Huazhong University of Science and Technology, Wuhan, China; 6 Unit of Respiratory Infection and Immunity, Institute Pasteur of Shanghai, Chinese Academy of Sciences, Shanghai, China; 7 Shanghai Key Laboratory of Lung Inflammation and Injury, Shanghai, China

**Keywords:** Asthma, Cell death and immune response

## Abstract

Polymerase 1 and transcript release factor (PTRF, encoding by *Cavin-1*) regulates interleukin 33 (IL-33) release, which is implicated in asthma development. Z-DNA binding protein 1 (ZBP1)-sensing Z-RNAs induces necroptosis which causes inflammatory diseases. House dust mite (HDM) is the major source of allergen in house dust and is strongly associated with the development of asthma. Whether PTRF via IL-33 and ZBP1 mediates HDM-induced macrophage necroptosis and airway inflammation remains unclear. Here, we found that deficiency of PTRF could reduce lung IL-33, ZBP1, phosphor-receptor-interacting protein kinase 3 (p-RIPK3), and phosphor-mixed lineage kinase domain-like (p-MLKL) (necroptosis executioner), and airway inflammation in an HDM-induced asthma mouse model. In HDM-treated macrophages, ZBP1, p-RIPK3, and p-MLKL levels were markedly increased, and these changes were reversed by deletion of *Cavin-1*. Deletion of *Il33* also reduced expression of ZBP1, p-RIPK3, and p-MLKL in HDM-challenged lungs. Moreover, IL-33 synergizing with HDM boosted expression of ZBP1, p-RIPK3, and p-MLKL in macrophages. In bronchial epithelial cells rather than macrophages and vascular endothelial cells, PTRF positively regulates IL-33 expression. Therefore, we conclude that PTRF mediates HDM-induced macrophage ZBP1/necroptosis and airway inflammation, and this effect could be boosted by bronchial epithelial cell-derived IL-33. Our findings suggest that PTRF-IL33-ZBP1 signaling pathway might be a promising target for dampening airway inflammation.

## Introduction

Asthma is a widespread airway disorder leading to wheezing, shortness of breath, chest tightness, and cough. Asthma is characterized by chronic airway inflammation, triggering processes such as airway hyperresponsiveness (AHR), mucus production, and remodeling of the airway wall. Asthma is a common chronic disease among both adults and children in the United States, affecting 25 million people and resulting in nearly one-half million hospitalizations annually [1]. Only some patients respond well to the medications and strategies currently used in the clinic. Asthma results from a complex interaction between structural and immune cells after exposure to specific environmental triggers [2]. Therefore, further understanding the mechanism underlying asthma may allow physicians to decide the best treatment for asthmatic patients.

Polymerase 1 and transcript release factor (PTRF), coded by *Cavin-1*, is a cytoplasmic protein containing a putative leucine zipper, a nuclear localization signal, and a PEST (amino acid sequence enriched in proline (P), glutamic acid (E), serine (S), and threonine (T)) domain [3]. Ubiquitously expressed in multiple tissues, including the lung, PTRF was initially identified as regulating transcription by interacting with RNA polymerase 1 and dissociating the paused transcription complex involving transcription termination factor 1 (TTF-1). PTRF has been suggested to be an essential structural component of caveolae. Caveolae are vital plasma membrane sensors that can respond to plasma membrane stresses and remodel the extracellular environment [4]. Moreover, accumulating evidence has shown that caveolae are present in various cells in the lung and interact with other proteins, receptors, and ion channels, potentially affecting normal and disease processes such as contractility, inflammation, and fibrosis [5]. PTRF participates in some key pathways during the progression of many lung diseases. For example, PTRF plays a role in receptor tyrosine kinases (RTK)-mediated pro-survival signaling in lung adenocarcinomas [6], also PTRF is a protein biomarker for chronic obstructive pulmonary disease (COPD) [7]. In an ovalbumin (OVA)-induced asthma mouse model, PTRF is reported to have an inhibitory effect on IL-33 release [8].House dust mite (HDM) is the major source of allergen in house dust and is strongly associated with the development of asthma [9]. However, whether PTRF is involved in the development of HDM-induced allergic airway inflammation, and the detailed mechanisms remain unknown.

Necroptosis is a programmed lytic cell death process [10], which is involved in a variety of inflammatory processes [11], especially in asthma [12]. Z-DNA binding protein 1 (ZBP1), a stress granule-associated protein, is known to activate necroptosis via receptor-interacting protein kinase 3 (RIPK3), another RIP-homotypic interaction motif (RHIM)-containing protein [13]. Once activated, the RHIM of ZBP1 binds the RHIM of RIPK3, stimulating RIPK3 kinase activity, auto-phosphorylation, and oligomerization. Then, p-RIPK3 phosphorylates the necroptosis effector mixed lineage kinase domain-like (MLKL), forming the necroptotic pore and releasing highly inflammatory intracellular components into the surrounding milieu [14]. Whether PTRF regulates ZBP1-medited necroptosis is worthy of investigation.

As we know, IL-33 contributes to the activation of type 2 immunity cells, such as group 2 innate lymphoid cells (ILC2s), T helper 2 cells (Th2), macrophages, and eosinophils in the development of asthma. IL-33 is also an important pro-inflammatory cytokine released from necrotic cells. Our previous study found that PTRF phosphorylation status regulated IL-33 release and eventually affected asthma exacerbation [8]. Necroptosis directly induces the release of full-length biologically active IL-33 in an inflammatory disease model and in vitro [15]. Whether IL-33 affects ZBP1-medited necroptosis is unknown.

Therefore, the objectives of this study are to determine: (i) whether deficiency of PTRF would attenuate HDM-induced airway inflammation; (ii) Whether deficiency of PTRF affects *Zbp1* expression and necroptosis executioners (p-RIPK3 and p-MLKL), IL-33 in HDM-challenged lungs; (iii) Whether deficiency of PTRF affects ZBP1, p-RIPK3, and p-MLKL levels in HDM-challenged macrophages; and (iv) whether IL-33 synergizes with HDM to boost ZBP1, p-RIPK3, and p-MLKL signaling in HDM-challenged macrophages. Ultimately, we will identify whether PTRF-IL33-ZBP1 signaling would mediate necroptosis in macrophages, which contributes to HDM-induced airway inflammation.

## Results

### Deficiency of PTRF ameliorates HDM-induced airway inflammation

**Fig. 1:**
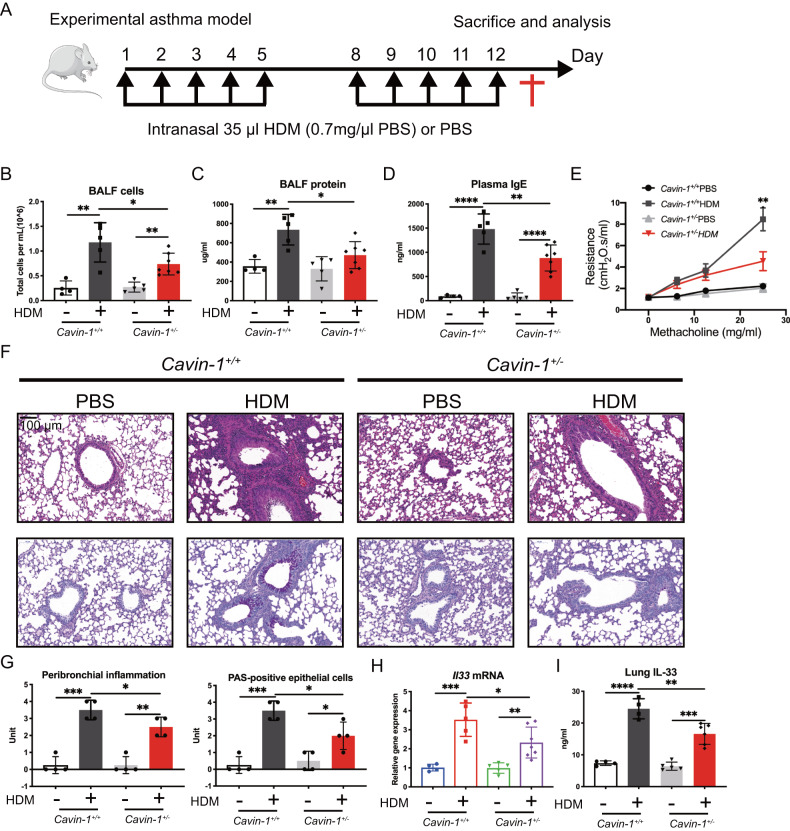
Deficiency of *Cavin-1* ameliorates HDM-induced airway inflammation. **A** Experimental scheme for HDM-induced mouse model of asthma. **B** Total cell counts in bronchoalveolar lavage fluid (BALF). **C** Total protein levels in BALF. **D** Plasma immunoglobulin E (IgE) levels were measured by ELISA. **E** Airway resistance index responded to increasing doses of methacholine. **F** Histological examination for lung paraffin sections of mice stained with hematoxylin and eosin (HE, upper panel) and periodic acid Schiff (PAS, lower panel). Scale bar, 100 μm. **G** Inflammation scores of lung tissues and analysis of PAS-positive cells. **H** Expression of IL-33 mRNA in lungs of *Cavin-1*^*+/+*^ and *Cavin-1*^*+/–*^ mice. **I** IL-33 protein levels were detected by ELISA in lung homogenates of *Cavin-1*^*+/+*^ and *Cavin-1*^*+/–*^ mice. Each point represents an individual mouse. **B**, **C**, **D**, **H**, **I**, data were means ± SD with *n* = 4–7 mice in per group. **G** data were means ± SD with *n* = 4 mice in per group. *P < 0.05, **P < 0.01, ***P < 0.001, ****P < 0.0001 as calculated by two-tailed unpaired student’s *t* test, corrected by one-way ANOVA with Turkey post-hoc test.

The genotyping protocols to identify *Cavin-1*^*+/+*^*, Cavin-1*^*+/–*^, and *Cavin-1*^*–/–*^ mice and confirmation of PTRF expression at both mRNA and protein levels in these mice were shown in Supplementary Fig. 1A–C. *Cavin-1*^*–/–*^ mice suffered from very low birth rate and severe growth problems, so we only obtained 3 homozygotes during 3 years of breeding. These 3 *Cavin-1*^*–/–*^ mice were used for HDM-challenged lung RNAseq analysis. Alternatively, *Cavin-1*^*+/–*^ mice were used in our routine experiments. To investigate the potential role of PTRF in regulating airway inflammation induced by HDM, we established a mouse asthma model by intranasal administrations of HDM (Fig. 1A). In *Cavin-1*^*+/+*^ mice, HDM treatment significantly increased total cells and protein in bronchoalveolar lavage fluid (BALF) compared with PBS-treated mice (Fig. 1B, C). We also found plasma immunoglobulin E (IgE) was increased in the asthma model (Fig. 1D). BALF inflammatory parameters and plasma IgE antibody titers were reduced in HDM-treated *Cavin-1*^*+/−*^ mice compared with HDM-treated *Cavin-1*^*+/+*^ littermates. Airway resistance, an indicator of AHR, was also significantly decreased in HDM-treated *Cavin-1*^+/−^ mice compared with HDM-treated *Cavin-1*^*+/+*^ littermates (Fig. 1E). Further histopathological examination of lung sections with hematoxylin and eosin (HE) and periodic acid–schiff (PAS) staining revealed that peribronchial inflammatory cell infiltration and mucus expression were reduced in HDM-treated *Cavin-1*^*+/−*^ mice compared to HDM-treated *Cavin-1*^*+/+*^ mice (Fig. 1F, G). Lung IL-33 at both mRNA and protein levels were decreased in HDM-treated *Cavin-1*^*+/−*^ mice compared to HDM-treated *Cavin-1*^*+/+*^ mice (Fig. 1H, I). The findings suggest that PTRF is causative factor for the development of HDM-induced airway inflammation and positively regulates IL-33 expression.

### Deficiency of PTRF reduces lung recruitment of eosinophils, macrophages, Th2, and Th17 cells in HDM-challenged mice

**Fig. 2:**
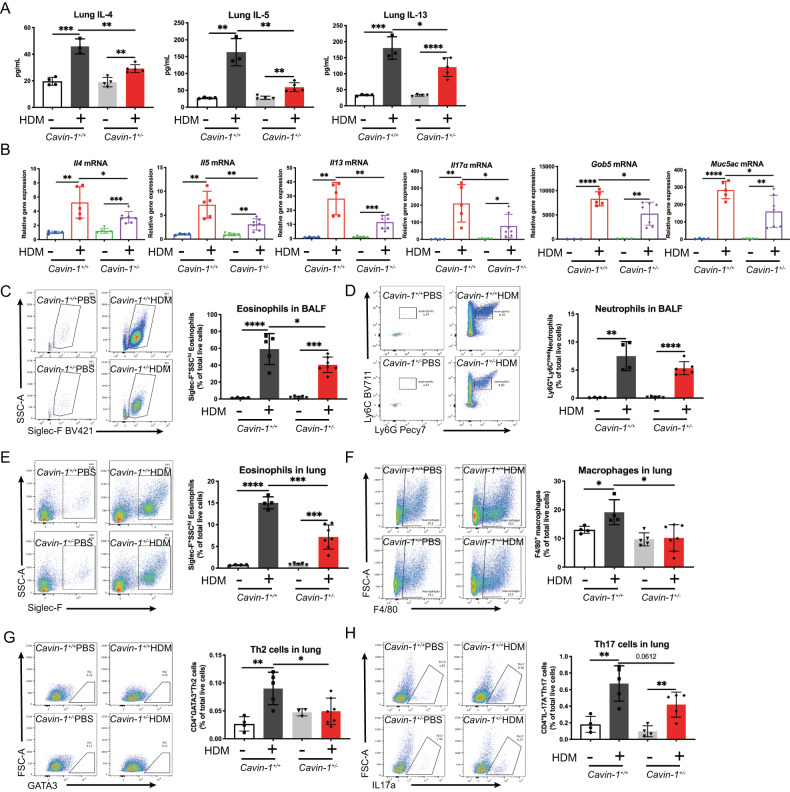
Deficiency of PTRF reduces lung recruitment of eosinophils, macrophages, Th2, and Th17 cells in HDM-challenged mice. **A** IL-4, IL-5 and IL-13 protein levels were measured by ELISA in lung lobe homogenates. **B** *Il4, Il5, Il13, Il17a, Gob5, Muc5ac* mRNAs were assessed by RT-qPCR (normalized to GAPDH). **C**-**D** Flow cytometric analysis of frequencies of Siglec-F^+^SSC-A^hi^ eosinophils and Ly6G^+^Ly6C^med^ neutrophils in BALF. **E**-**H** Flow cytometry analysis of cell population in the lung, including Siglec-F^+^SSC-A^hi^ eosinophils, F4/80^+^ macrophages, CD4^+^GATA3^+^ Th2 cells and CD4^+^IL-17A^+^ Th17 cells. (*n* = 3–7 mice in each group) Data were represented as means ± SD. **A** *n* = 3–5 mice; (**B**, **C**, **E**), *n* = 4–7 mice; (**D**, **F**), *n* = 4–6 mice; (**G**), *n* = 3–7 mice; (**H**) *n* = 4–5 mice. *P < 0.05, **P < 0.01, ***P < 0.001, ****P < 0.0001 as calculated by two-tailed unpaired student’s *t* test, corrected by one-way ANOVA with Turkey post-hoc test.

The ELISA analysis showed that in HDM-treated *Cavin-1*^*+/+*^ lungs, the levels of inflammatory cytokines such as IL-4, IL-5, and IL-13 were increased versus vehicle-treated *Cavin-1*^*+/+*^ lungs, and this change was reversed in HDM-challenged *Cavin-1*^*+/–*^ lungs (Fig. 2A). Quantitative PCR analysis showed that expression of lung *Il4, Il5, Il13, Il17a, Gob5*, and *Muc5ac* in HDM-challenged *Cavin-1*^*+/–*^ mice was lower than that in HDM-challenged *Cavin-1*^*+/+*^ mice (Fig. 2B). Flow cytometric analysis demonstrated that eosinophils in both BALF and lung were increased in HDM-challenged *Cavin-1*^*+/+*^ mice compared to vehicle-challenged *Cavin-1*^*+/+*^ mice, and this change was reversed in HDM-challenged *Cavin-1*^*+/-*^ mice (Fig. 2C, E). The BALF neutrophils did not differ in above two groups (Fig. 2D). The lung macrophages (Fig. 2F), Th2 (Fig. 2G), and Th17 (Fig. 2H) cells were higher in HDM-challenged *Cavin-1*^*+/+*^ mice compared to vehicle-challenged *Cavin-1*^*+/+*^ mice; however, these parameters were lower in HDM-challenged *Cavin-1*^*+/-*^ mice compared to HDM-challenged *Cavin-1*^*+/+*^ mice. These data strongly support that PTRF is a mediator of HDM-induced airway inflammation.

### PTRF positively regulates ZBP1, necroptosis, IL-33 during HDM-induced airway inflammation

**Fig. 3:**
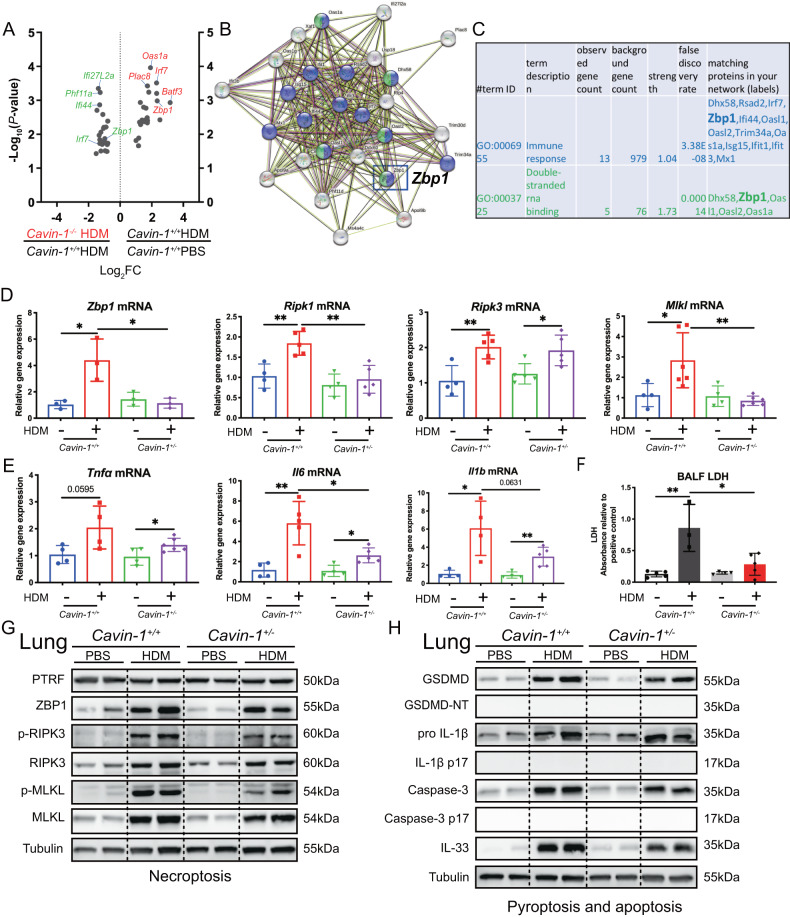
PTRF augments ZBP1, necroptosis, IL-33 during HDM-induced airway inflammation. **A** Volcano plot of downregulated gene expression in lungs from HDM-treated *Cavin-1*^*–/–*^ mice versus HDM-treated *Cavin-1*^*+/+*^ mice and upregulated gene expression in lungs from HDM-treated *Cavin-1*^*+/+*^ mice versus PBS-treated *Cavin-1*^*+/+*^ mice. **B**, **C** Protein interaction network, GO analysis of immune response and double-stranded RNA binding genes in above groups. Results of RNA-sequence represented one experiment including 11 samples totally (*n* = 2–3 mice in each group). **D** *Zbp1, Ripk1, Ripk3, and Mlkl* mRNAs were assessed by RT-qPCR (normalized to GAPDH). **E** *Tnfα, Il6, and Il1β* mRNAs were assessed by RT-qPCR (normalized to GAPDH). **F** Lactate dehydrogenase (LDH) activity measured at an optical density of 490 nm (OD490) in BALF samples (*n* = 3–5 mice). **G** Western blot analysis of PTRF, ZBP1 and necroptosis components in the lungs collected from PBS or HDM treated *Cavin-1*^*+/+*^ and *Cavin-1*^*+/*–^ mice. Necroptosis activation was indicated by the phosphorylation of receptor interacting protein kinase 3 (p-RIPK3) and mixed lineage kinase domain-like pseudokinase (p-MLKL). **H** Western blot analysis of pyroptosis and apoptosis activation markers after HDM exposure in the lungs collected from PBS or HDM treated *Cavin-1*^*+/+*^ and *Cavin-1*^*+/–*^ mice. Pyroptosis activation was assessed by immunoblotting of cleaved GSDMD (35 kDa) and mature IL-1β (17 kDa). Apoptosis activation was determined by immunoblotting of executioner caspase3 (17 kDa). IL-33 in the lung tissues was also detected with immunoblotting. In (**G**, **H**), tubulin was used as a loading control for immunoblot analysis and molecular weight marker sizes were indicated on the right (kDa). Data were from three independent experiments (*n* = 3–6 mice in each group) and represented as means ± SD. *P < 0.05, **P < 0.01 as calculated by two-tailed unpaired student’s *t* test, corrected by one-way ANOVA with Turkey post-hoc test.

By RNA-seq data analysis, we found that HDM challenge significantly upregulated lung immune response (*Dhx58, Rsad2, Irf7, Zbp1, Ifi44, Oasl1, Oasl2, Trim34a, Oas1a, Isg15, Ifit1, Ifit3, and Mx1)* and double stranded RNA binding (*Dhx58, Zbp1, Oasl1, Oasl2, and Oas1a*) genes, especially *Zbp1* (Fig. 3A–C). However, these genes were significantly downregulated in HDM-challenged *Cavin-1*^–/–^ lungs *compared to* HDM-challenged *Cavin-1*^*+/+*^ lungs. Considering that ZBP1 plays critical roles during both DNA and RNA virus infection as the sensor responsible for triggering PANoptosis (pyroptosis, apoptosis, and necroptosis) [16], we measured *Zbp1* and necroptosis-related genes in either vehicle or HDM challenged *Cavin-1*^*+/+*^ and *Cavin-1*^*+/–*^ lungs.

RT-PCR analysis showed that HDM treatment upregulated *Zbp1, Ripk1, Ripk3*, and *Mlkl* mRNA levels in the *Cavin-1*^*+/+*^ lungs. However, these genes were markedly reduced in HDM-treated *Cavin-1*^*+/*–^ lungs (Fig. 3D). Concurrently, the lung proinflammatory cytokine (TNF-α, IL-6, and IL-1β) genes and lactate dehydrogenase (LDH, an index of cell death) levels were lower in HDM-treated *Cavin-1*^*+/*–^ mice compared to HDM-treated *Cavin-1*^*+/+*^mice (Fig. 3E, F). For confirmation, we performed Western blotting of lung homogenates to assess typical signaling pathways in necroptosis, pyroptosis, and apoptosis. We found that expression of ZBP1, p-RIPK3, and p-MLKL in HDM-treated *Cavin-1*^*+/+*^ lungs were significantly increased than that in HDM-treated *Cavin-1*^*+/*–^ lungs (Fig. 3G). No significant difference was found in N-terminal Gasdermin D (GSDMD) and pro-IL-1β cleavage (pyroptosis markers), and cleaved caspase-3 (an index of apoptosis) between HDM-treated *Cavin-1*^*+/+*^ lungs and HDM-treated *Cavin-1*^*+/–*^ lungs (Fig. 3H). Importantly, lung IL-33 levels were lower in HDM-treated *Cavin-1*^*+/–*^ lungs than that in HDM-treated *Cavin-1*^*+/+*^ lungs (Fig. 3H). These findings support that PTRF is a positive regulator of ZBP1, necroptosis, IL-33 during the HDM-induced airway inflammation.

### HDM challenge increases expression of PTRF, ZBP1, and necroptosis in macrophages (Raw264.7)

**Fig. 4:**
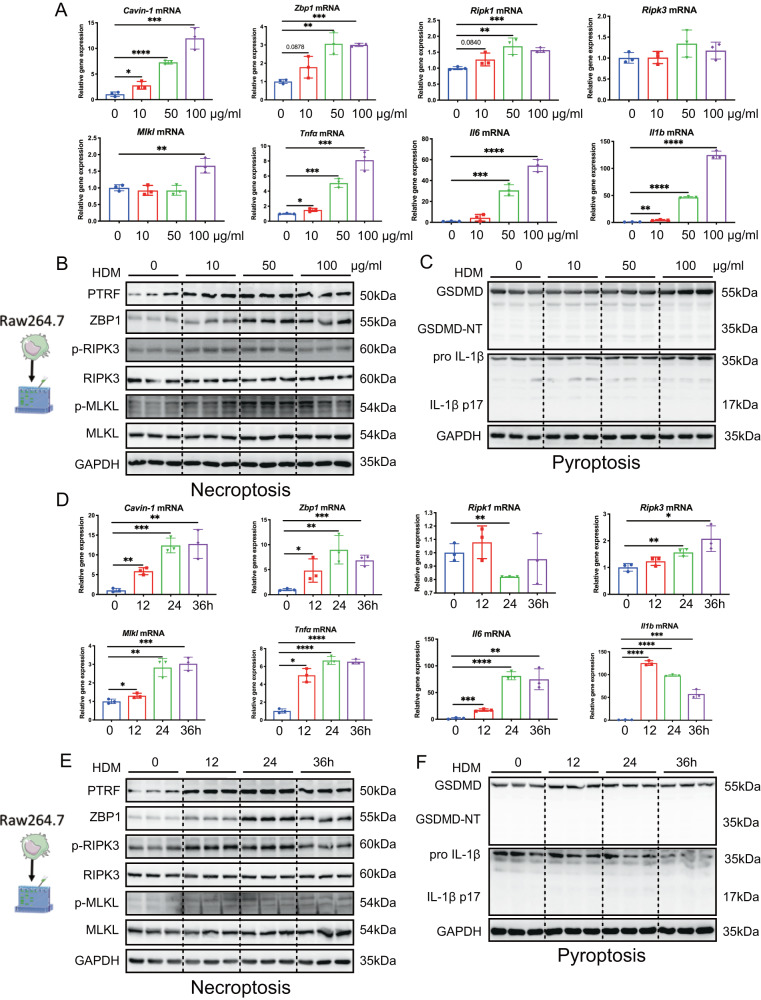
HDM challenge increases expression of PTRF, ZBP1, and necroptosis in macrophages (Raw264.7). **A**–**C** The Raw264.7 cells were stimulated with indicated concentration of HDM for 24 h. **D**–**F** The Raw264.7 cells were stimulated with HDM (50 μg/ml) for indicated time intervals. **A**, **D** *Cavin-1, Zbp1, Ripk1, Ripk3, Mlkl*, *Tnfα, Il6* and *Il1β* mRNAs were assessed by RT-qPCR (normalized to GAPDH). **B**, **E** Western blot analysis of PTRF, ZBP1 and necroptosis components in cell lysates. Necroptosis activation was indicated by p-RIPK3 and p-MLKL. **C**, **F** Western blotting analysis of pyroptosis markers cleaved GSDMD and mature IL-1β after HDM exposure in cell lysates. Data represented as means ± SD and *n* = 3 biological replicates in each group, *P < 0.05, **P < 0.01, ***P < 0.001, ****P < 0.0001 as calculated by two-tailed unpaired student’s *t* test.

We challenged mouse macrophages (Raw264.7) with different concentration of HDM. RT-PCR analysis revealed that HDM increased expression of *Cavin-1*, *Zbp1, Ripk1, Tnfa, Il6, and Il1b* (Fig. 4A). Immunoblot analysis showed that HDM challenge also upregulated expression of PTRF and ZBP1, phosphorylation of RIPK3 and MLKL (Fig. 4B); however, HDM treatment did not affect cleavage of GSDMD and pro-IL-1β (Fig. 4C). The optimal concentration of HDM was 50 μg/ml to induce macrophage expression of PTRF and ZBP1, and necroptosis executioners. These findings suggest that HDM could induce necroptosis in macrophages.

To study time-dependent effect of HDM challenge, we then treated Raw264.7 with HDM at 50 μg/ml for 0, 12, 24, and 36 h. We found that HDM increased the mRNA levels of *Cavin-1*, *Zbp1*, and necroptosis-related genes *Ripk3* and *Mlkl* at 24 h after HDM challenge. The same pattern was also in *Tnfα, Il6, and Il1b* expression (Fig. 4D). Consistent with mRNA analysis, HDM treatment also increased the expression of PTRF and ZBP1, phosphorylation of RIPK3 and MLKL at 24 h (Fig. 4E), but did affect cleavage of GSDMD and pro-IL-1β (Fig. 4F). These findings confirm that HDM is able to trigger necroptosis in macrophages by upregulating necrotizing signaling pathway molecules.

We repeated the above experiments in bronchial epithelial cells (BEAS-2B) and vascular endothelial cells (HUVEC) using the same experimental protocols. We found that HDM challenge could increase IL-33 expression at concentration of 50 and 100 μg/ml in bronchial epithelial cells. Knockdown of *Cavin-1* could reduce IL-33 expression in HDM-treated bronchial epithelial cells, suggesting that PTRF positively regulates IL-33 *e*xpression in HDM-challenged bronchial epithelial cells (Supplementary Fig. 2A, B). HDM and knockdown of *Cavin-1* did not alter IL-33 expression in HUVEC (Supplementary Fig. 2C, D). HDM and knockdown of *Cavin-1* did not affect expression of PTRF and ZBP1, and p-RIPK3 and p-MLKL in both bronchial epithelial and vascular endothelial cells either (Supplementary Fig. 2A–D).

### Knockdown of *Cavin-1* in Raw264.7 macrophages attenuates HDM-triggered ZBP1/necroptosis signaling

**Fig. 5:**
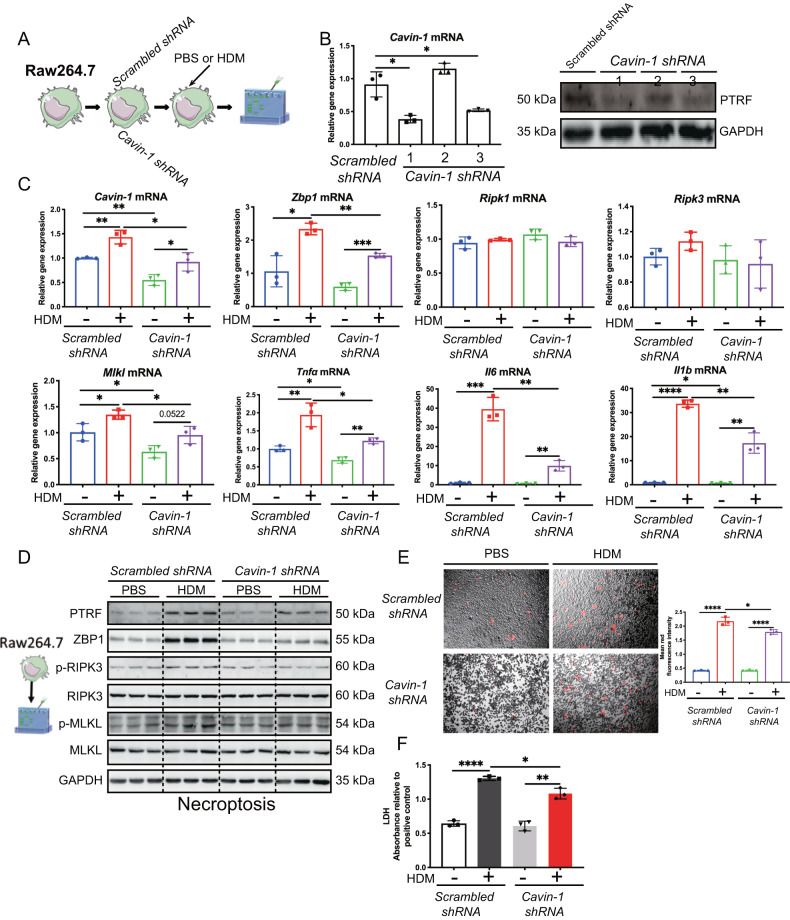
Knockdown of *Cavin-1* in Raw264.7 macrophages attenuates HDM-triggered ZBP1/necroptosis signaling. **A** The Raw264.7 cells treated with scrambled shRNA or *Cavin-1* shRNA were stimulated with HDM (50 μg/ml) for 24 h. **B** Raw264.7 were transfected with either scrambled or *Cavin-1* shRNAs and Western blot was performed for the protein expression of PTRF in cell lysates. **C** *Cavin-1, Zbp1, Ripk1, Ripk3, Mlkl*, *Tnfα, Il6* and *Il1b* mRNAs were assessed by RT-qPCR (normalized to GAPDH). **D** Western blot analysis of PTRF, ZBP1 and necroptosis components in cell lysates. Necroptosis activation was indicated by p-RIPK3 and p-MLKL. **E** Real-time analysis of cell death in Raw 264.7 using the PI staining after treated with HDM for 24 h. The original magnification is ×10. Quantification of mean fluorescence intensity of PI staining cells. **F** LDH activity measured at OD490 in cell supernatant. Data were means ± SD and *n* = 3 biological replicates in per group, *P < 0.05, **P < 0.01, ***P < 0.001, ****P < 0.0001 as calculated by two-tailed unpaired student’s *t* test, corrected by one-way ANOVA with Turkey post-hoc test.

We next investigated whether PTRF would regulate ZBP1 and necroptosis in vitro. We built up 3 *Cavin-1 shRNA* to silence *Cavin-1* in Raw264.7 macrophages. More than 50% PTRF was deleted at both mRNA and protein levels using the NO.1 construct of *Cavin-1 shRNA* (Fig. 5A, B). We treated the Raw264.7 macrophages with the NO.1 construct of *Cavin-1 shRNA* and its scrambled counterpart, followed by challenging the cells with HDM. By RT-PCR analysis, we found that *Zbp1*, necroptosis-related *Mlkl*, *Tnfα, Il6, and Il1b* at mRNA levels were significantly upregulated in HDM-challenged *scrambled shRNA* transfected cells. In contrast, these genes were markedly reduced in HDM-challenged *Cavin-1 shRNA* transfected cells compared to HDM-challenged *scrambled shRNA* transfected cells (Fig. 5C). By immunoblotting analysis, we found that HDM challenge upregulated PTRF, ZBP1, p-RIPK3, and p-MLKL in *scrambled shRNA* transfected cells; however, this change was reversed in *Cavin-1 shRNA* transfected cells (Fig. 5D). We also detected cell membrane integrity with propidium iodide (PI) staining by microscopy and found that cell death induced by HDM was quantitatively reduced in *Cavin-1 shRNA* transfected cells compared with *scrambled shRNA* transfected cells (Fig. 5E). LDH was also increased in HDM-challenged *scrambled shRNA* transfected cells, and this change was also attenuated in HDM-challenged *Cavin-1 shRNA* transfected cells (Fig. 5F). These findings further support that PTRF positively regulates ZBP1/necroptosis in HDM-challenged macrophages.

### Knockdown of *Zbp1* in Raw264.7 macrophages attenuates HDM-triggered necroptosis signaling

**Fig. 6:**
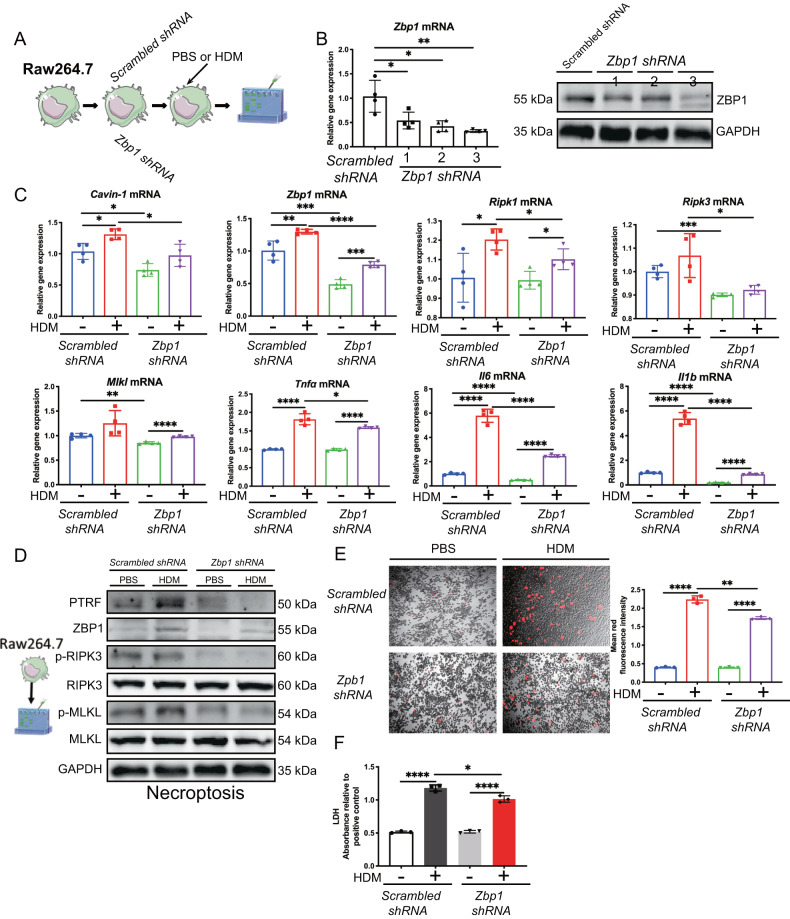
Knockdown of *Zbp-1* in Raw264.7 macrophages attenuates HDM-triggered necroptosis signaling. **A** The Raw264.7 cells treated with scrambled shRNA or *Zbp-1* shRNA were stimulated with HDM (50 μg/ml) for 24 h. **B** Raw264.7 were transfected with either scrambled or *Zbp-1* shRNAs and Western blot was performed for the protein expression of ZBP1 in cell lysates. **C** *Cavin-1, Zbp1, Ripk1, Ripk3, Mlkl*, *Tnfα, Il6* and *Il1b* mRNAs were assessed by RT-qPCR (normalized to GAPDH). **D** Western blot analysis of PTRF, ZBP1 and necroptosis components in cell lysates. Necroptosis activation was indicated by p-RIPK3 and p-MLKL. **E** Real-time analysis of cell death in Raw 264.7 using the PI staining after treated with HDM for 24 h. The original magnification is ×10. Quantification of mean fluorescence intensity of PI staining cells. **F** LDH activity measured at OD490 in cell supernatant. Data were means ± SD and *n* = 3 biological replicates in per group, *P < 0.05, **P < 0.01, ***P < 0.001, ****P < 0.0001 as calculated by two-tailed unpaired student’s *t* test, corrected by one-way ANOVA with Turkey post-hoc test.

We further explored whether ZBP1 would regulate necroptosis in vitro. We built up 3 *Zbp1 shRNA* to silence *Zbp1* in Raw264.7 macrophages. More than 50% ZBP1 was deleted at both mRNA and protein levels using the NO.3 construct of *Zbp1 shRNA* (Fig. 6A, B). We treated the Raw264.7 macrophages with the NO.3 construct of *Zbp1 shRNA* and its scrambled counterpart, followed by challenging the cells with HDM. By RT-PCR analysis, we found that necroptosis-related *Ripk1*, Ripk3, *Tnfα, Il6, and Il1b* at mRNA levels were significantly upregulated in HDM-challenged *scrambled shRNA* transfected cells. In contrast, these genes were markedly reduced in HDM-challenged *Zbp1 shRNA* transfected cells compared to HDM-challenged *scrambled shRNA* transfected cells (Fig. 6C). We observed that necroptosis molecules ZBP1 and p-MLKL were upregulated in *scrambled shRNA* transfected cells following HDM stimulation; however, this change was reversed in *Zbp1 shRNA* transfected cells (Fig. 6D). We also found that cell death induced by HDM stimulation was reduced in *Zbp1 shRNA* transfected cells compared to *scrambled shRNA* transfected cells with PI staining (Fig. 6E). LDH was also increased in HDM-challenged *scrambled shRNA* transfected cells, and this change was also attenuated in HDM-challenged *Zbp1 shRNA* transfected cells (Fig. 5F). Taken together, these results imply that ZBP1 regulates necroptosis in HDM-challenged macrophages.

### Deletion of *Cavin-1* in BMDMs attenuates HDM-triggered ZBP1/necroptosis

**Fig. 7:**
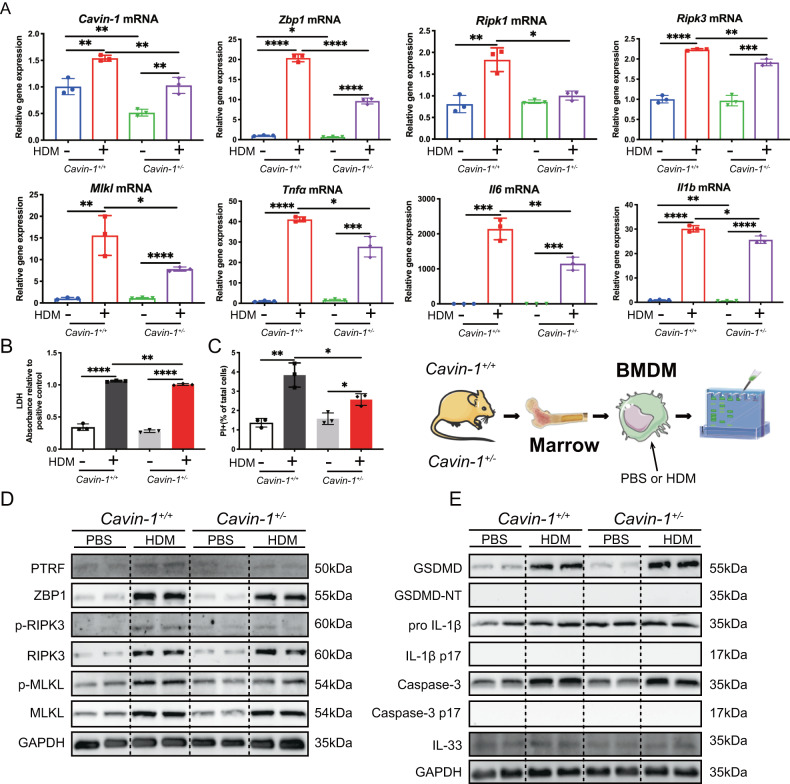
Deletion of *Cavin-1* in BMDMs attenuates HDM-triggered ZBP1/necroptosis. The BMDMs isolated and developed from the bone marrows of *Cavin-1*^*+/*–^ and *Cavin-1*^*+/*–^ mice were stimulated with HDM (50 μg/ml) for 24 h. **A** *Cavin-1, Zbp1, Ripk1, Ripk3, Mlkl*, *Tnfα, Il6* and *Il1b* mRNAs were assessed by RT-qPCR (normalized to GAPDH). **B** LDH activity measured at OD490 in cell supernatant. **C** The necroptosis of BMDMs treated with HDM (50 μg/ml) for 24 h were analyzed by PI staining followed by flow cytometry analysis. **D** Western blot analysis of PTRF, ZBP1 and necroptosis components in BMDMs lysates. Necroptosis activation was indicated by p-RIPK3 and p-MLKL. **E** Western blotting analysis of pyroptosis markers cleaved GSDMD and mature IL-1β and apoptosis executioner cleaved caspase3 in BMDMs lysates. IL-33 in the BMDMs was also detected with immunoblotting. Data were means ± SD and *n* = 3 biological replicates in each group, *P < 0.05, **P < 0.01, ***P < 0.001, ****P < 0.0001 as calculated by two-tailed unpaired student’s *t* test, corrected by one-way ANOVA with Turkey post-hoc test.

For confirmation, we decided to use bone marrow-derived macrophages (BMDMs) to repeat our experiments. *Cavin-1*^*+/+*^ and *Cavin-1*^*+/–*^ BMDMs were developed and treated with HDM 50 μg/mL for 24 h. By RT-PCR analysis, we found that *Cavin-1*, *Zbp1, Ripk1, Ripk3, Mlkl, Tnfα, Il6, and Il1b* genes were significantly upregulated in HDM-challenged *Cavin-1*^*+/+*^ BMDMs; however, this change was reversed in HDM-challenged *Cavin-1*^*+/–*^ BMDMs (Fig. 7A). Furthermore, LDH and PI positive cells (by flow cytometric analysis) were increased in HDM-challenged *Cavin-1*^*+/+*^ BMDMs, and this change was also attenuated in HDM-challenged *Cavin-1*^*+/–*^ BMDMs (Fig. 7B, C). By immunoblotting analysis, we found that PTRF, ZBP1, and necroptosis-executioners, p-RIPK3 and p-MLKL, were markedly upregulated in HDM-challenged *Cavin-1*^*+/+*^ BMDMs, and this change was reduced in HDM-challenged *Cavin-1*^*+/-*^ BMDMs (Fig. 7D). These findings confirm that PTRF could induce ZBP1/necroptosis in macrophages. The cleavage of GSDMD, Pro-IL-1β, and activated caspase 3 was not different between HDM-challenged *Cavin-1*^*+/+*^ and *Cavin-1*^*+/–*^ BMDMs. Although HDM slightly increased IL-33 expression, but *Cavin-1* knockdown did not affect the expression of IL-33 in HDM-challenged BMDMs (Fig. 7E).

### Loss of *Il33* attenuates HDM-triggered ZBP1/necroptosis in a pattern similar to PTRF

**Fig. 8:**
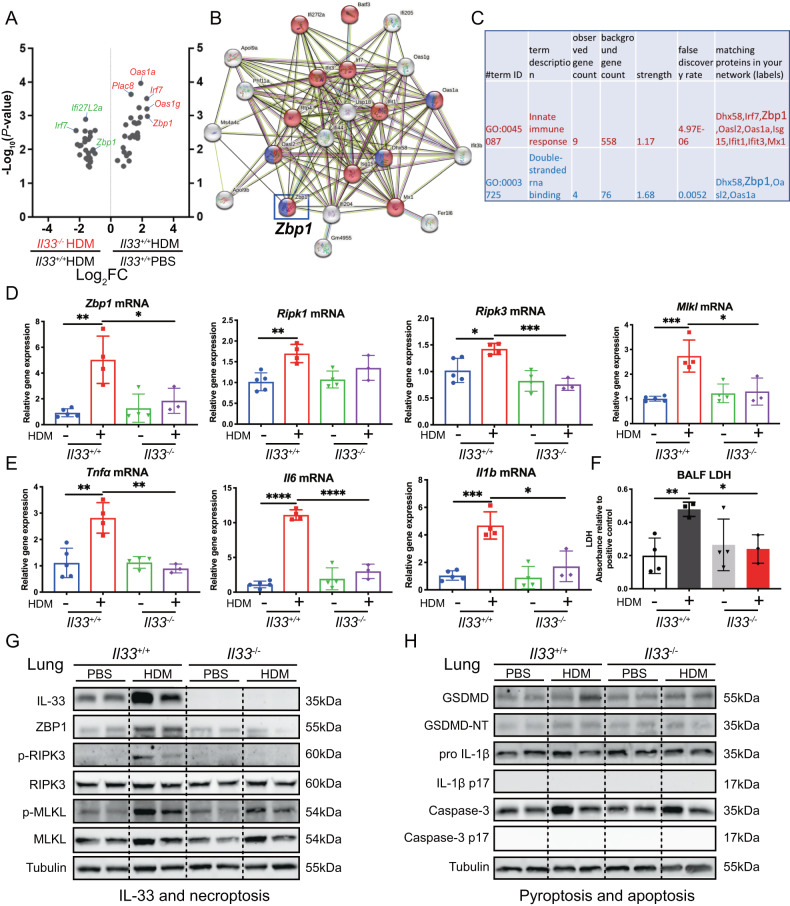
Loss of *IL33* attenuates HDM-triggered ZBP1/necroptosis in a pattern similar to PTRF. **A** Volcano plot of downregulated gene expression in lungs from HDM-treated *Il33*^*–/–*^ mice versus HDM-treated *Il33*^*+/+*^ mice and upregulated gene expression in lungs from HDM-treated *Il33*^*+/+*^ mice versus PBS-treated *Il33*^*+/+*^ mice. **B**, **C** Protein interaction network, GO analysis of immune response and double-stranded RNA binding genes in above groups. Results of RNA-sequence represented one experiment including 12 samples totally (*n* = 3 mice in each group). **D** *Zbp1, Ripk1, Ripk3, Mlkl* mRNAs were assessed by RT-qPCR (normalized to GAPDH). **E** *Tnfα, Il6, Il1b* mRNAs were assessed by RT-qPCR (normalized to GAPDH). **F** LDH activity measured at OD490 in BALF samples. **G** Western blot analysis of IL-33, ZBP1, and necroptosis components p-RIPK3, p-MLKL in the lung tissues of HDM induced mouse model with *Il33*^–/–^ and *Il3*^*+/+*^ mice. **H** Western blotting analysis of pyroptosis cleaved GSDMD, mature IL-1β and apoptosis executioner cleaved caspase3 after HDM exposure in lung tissues of *Il33*^–/–^ and *Il3*^*+/+*^ mice. In (**G**, **H**), tubulin was used as a loading control for immunoblot analysis and molecular weight marker sizes were indicated on the right (kDa). Data are means ± SD and *n* = 3–5 mice in each group, *P < 0.05, **P < 0.01, ***P < 0.001, ****P < 0.0001 as calculated by two-tailed unpaired student’s *t* test, corrected by one-way ANOVA with Turkey post-hoc test.

As shown in Supplementary Fig. 3, deletion of *Il33* reduced HDM-induced airway inflammation. To study whether IL-33 regulates *Zbp1* expression, we performed RNAseq analysis. We found that HDM challenge significantly upregulated lung immune response (*Dhx58, Irf7, Zbp1, Oasl2, Oas1a, Isg15, Ifit1, Ifit3*, and *Mx1)* and double stranded RNA biding (*Dhx58, Zbp1, Oasl2*, and *Oas1a*) genes, especially *Zbp1* (Fig. 8A–C). However, these genes were significantly downregulated in HDM-challenged *Il33*^–/–^ lungs *compared to* HDM-challenged *Il33*^*+/+*^ lungs. We detected *Zbp1* and necroptosis-related genes in either vehicle or HDM challenged *Il33*^*+/+*^ and *Il33*^–*/–*^ lungs. RT-PCR analysis showed that HDM treatment upregulated *Zbp1, Ripk1, Ripk3*, and *Mlkl* mRNA levels in the *Il33*^*+/+*^ lungs. However, *Zbp1, Ripk3*, and *Mlkl* genes were markedly reduced in HDM-treated *Il33*^*–/–*^ lungs (Fig. 8D). Concurrently, the lung proinflammatory cytokine (TNF-α, IL-6, and IL-1β) genes and BALF LDH levels were lower in HDM-treated *Il33*^*–/–*^ mice compared to HDM-treated *Il33*^*+/+*^ mice (Fig. 8E, F). For confirmation, we performed Western blotting of lung homogenates to assess typical signaling pathways in necroptosis, pyroptosis, and apoptosis. We found that HDM challenge increased lung IL-33 and ZBP1 expression in *Il33*^*+/+*^mice, and these proteins were significantly downregulated in *Il33*^*–/–*^ mice. The expression of p-RIPK3 and p-MLKL in HDM-treated *Il33*^*+/+*^ lungs were significantly increased than that in HDM-treated *Il33*^*-/-*^ lungs (Fig. 8G). No significant difference was found in N-terminal GSDMD and pro-IL-1β cleavage (pyroptosis markers), and cleaved caspase-3 (an index of apoptosis) between HDM-treated *Il33*^*+/+*^ lungs and HDM-treated *Il33*^*+/*–^ lungs (Fig. 8H). These findings support IL-33, in a similar pattern to PTRF, positively regulating ZBP1 expression and necroptosis during HDM-induced airway inflammation.

### HDM and IL-33 synergistically increases p-RIPK3 and p-MLKL in Raw264.7 macrophages and BMDMs

**Fig. 9:**
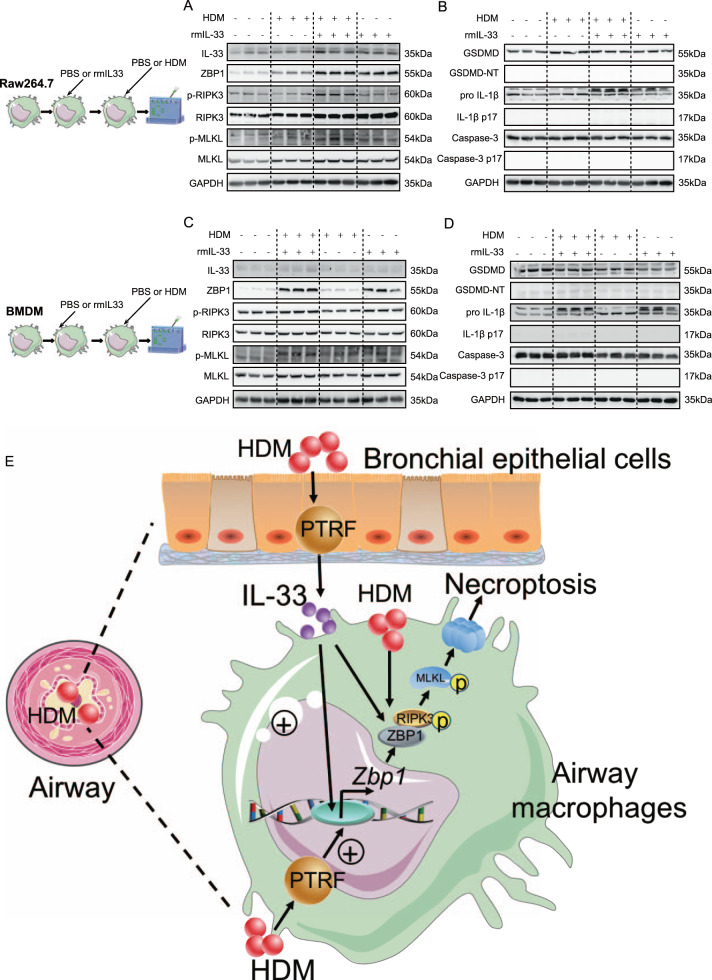
HDM and IL-33 synergistically increases p-RIPK3 and p-MLKL in Raw264.7 macrophages and BMDMs. Raw264.7 macrophages (**A**, **B**) and BMDMs from WT mouse (**C**, **D**) were treated with either PBS or IL-33 (rmIL-33, 50 μg/ml), and followed by either PBS or HDM (50 μg/ml) for 24 h. The cells were harvested for Western blotting analysis. **A**, **C** Western blot analysis of IL-33, ZBP1, and necroptosis components p-RIPK3, p-MLKL in cell lysates of Raw264.7 and BMDMs. **B**, **D** Western blotting analysis of pyroptosis markers cleaved GSDMD, mature IL-1β and apoptosis executioner cleaved caspase3 after HDM/rmIL-33 exposure in cell lysates. GAPDH was used as an internal control for immunoblot analysis and molecular weight marker sizes were indicated on the right (kDa). *n* = 3 biological replicates in each group. **E** PTRF positively regulates HDM-induced IL-33 expression in bronchial epithelial cells. Concurrently, PTRF increases ZBP1 expression and necroptosis signaling in HDM-challenged macrophages. Furthermore, bronchial epithelial cell-derived IL-33 works synergistically with HDM to enhance ZBP1 expression and necroptosis in HDM-challenged macrophages. Thus, the above effects contribute to HDM-induced macrophage necroptosis and airway inflammation.

We treated Raw264.7 macrophages and BMDMs from WT mouse with either PBS or IL-33, followed by PBS or HDM challenge. We found that IL-33 + HDM treated group could significantly increase ZBP1, p-RIPK3, and p-MLKL compared to IL-33 or HDM-treated group (Fig. 9A, C). However, HDM + IL-33 treatment did not affect cleavage of GSDMD, pro-IL-1β, and caspase 3 (Fig. 9B, D). These findings suggest that HDM and IL-33 synergistically increases promotes necroptosis in macrophages by upregulating p-RIPK3 and p-MLKL. In summary, as shown in Fig. 9E, PTRF positively regulates HDM-induced IL-33 expression in bronchial epithelial cells. Concurrently, PTRF increases ZBP1 expression and necroptosis signaling in HDM-challenged macrophages. Furthermore, bronchial epithelial cell-derived IL-33 works synergistically with HDM to enhance ZBP1 expression and necroptosis in HDM-challenged macrophages. The above processes contribute to airway inflammation.

## Discussion

In this study, we first demonstrated that deficiency of *Cavin-1* could attenuate HDM-induced airway inflammation. We then found that *Zbp1* expression and necroptosis executioners (p-RIPK3 and p-MLKL), and IL-33 were significantly decreased in *Cavin-1-*deficient HDM-challenged lungs. In macrophages, HDM challenge increased ZBP1, p-RIPK3, and p-MLKL levels, and these changes could be reversed by genetic knockdown or deletion of *Cavin-1*. More importantly, we found that ZBP1, p-RIPK3, and p-MLKL were reduced in *Il33-*deficient HDM-challenged lungs. PTRF positively regulates IL-33 in bronchial epithelial cells. In presence of IL-33, ZBP1, p-RIPK3, and p-MLKL were significantly increased in HDM-challenged macrophages.

PTRF, coded by *Cavin-1*, is a critical constituent of the caveolae structure on the plasma membrane [4]. Caveolae are flask-shaped invaginations of the plasma membrane present in most structural cells. The lungs express numerous caveolae and high levels of PTRF, which play important roles in pulmonary diseases such as lung cancer and pulmonary hypertension [6, 17]. Expression of PTRF in the airway smooth muscle is increased in an OVA-induced asthma model [18]. Partial loss of PTRF led to a greater AHR and potent type 2 immune responses during challenge phase of OVA-induced asthma model, without influencing the sensitization phase. Knockdown of PTRF in 16HBE led to a significantly increased level of IL-33 in cell culture supernatants in response to LPS or HDM [8]. In this study, we found that deletion of *Cavin-1* reduces HDM-induced airway inflammation and lung IL-33 at both mRNA and protein levels (Figs. 1 and 3H). Knockdown of *Cavin-1* also reduces IL-33 in HDM-induced BEAS-2B bronchial epithelial cells detected by Western blotting (Supplementary Fig. 2). These findings suggest that the modulating effect of PTRF on inflammation and IL-33 expression may depend on different cell lines or inducers of inflammation.

It is reported that necroptosis directly induces the release of nuclear IL-33 in its full-length form in an *Aspergillus* extract-induced asthma model. *Aspergillus* extract could trigger necroptosis and IL-33 release in L929 or HaCaT cells [15]. Here, we found that HDM can increase IL-33 expression in bronchial epithelial cells (Supplementary Fig. 2B) rather than macrophages (Fig. 7E) and vascular endothelial cells (Supplementary Fig. 2D). HDM could not induce necroptosis in the bronchial epithelial cells (Supplementary Fig. 2A). Hence, we speculate that HDM might work with the other proinflammatory cytokines (TNF-α, etc) to induce necroptosis and IL-33 release in the airway epithelial cells of HDM-challenged asthma mouse model. We have demonstrated that HDM-treated macrophages could produce TNF-α, and this effect could be attenuated by knockdown or deletion of *Cavin-1* (Figs. 5C and 7A). This notion was supported by airway epithelial cell necroptosis occurs in HDM-induced allergic inflammation mouse model [19].

Macrophages form the first line of defense against microbes and airborne particles through multiple functions, including phagocytosis, production of cytokines and chemokines, and antigen presentation. Emerging studies suggest ZBP1 is a pathogen sensor (for DNA and RNA) that regulates of cell death and inflammatory responses [20]. ZBP1 is abundantly expressed in macrophages and contributes to necroptosis [21]. ZBP1, which complexes with RIPK3 to trigger RIPK3-driven pathways, including trafficking and oligomerization of phosphorylated MLKL at the cell membrane results in cell lysis, characteristic of necroptotic cell death [22]. Interestingly, our HDM-challenged lung RNAseq analysis showed that effect of deletion of *Cavin-1* on immune response and double-stranded RNA-binding genes, especially *Zbp1*, has the same pattern as deletion of *ll33* (Figs. 3A–C and 8A–C). Western blotting analysis also showed that deletion of *Cavin-1* manifested as deletion of *Il33* could reduce ZBP1, p-RIPK3, and p-MLKL signaling pathway (Figs. 3G and 8G). Thus, we conclude that both PTRF and IL-33 positively regulate ZBP1-necroptosis during HDM-induced airway inflammation. More importantly, we confirmed that knockdown or deletion of *Cavin-1* could reduce HDM-induced necroptosis in HDM-challenged macrophages (Figs. 5C and 7A).

In our study, we also found that airway inflammation and necroptosis markers were decreased in *Il33*^–*/–*^ asthma mice, while co-exposure to HDM + rmIL-33 synergistically increased the ZBP1/necroptosis in Raw264.7 cells and BMDMs. We assumed that PTRF upregulated the expression of IL-33 in bronchial epithelial cells, which may contribute to HDM-promoted inflammation through upregulating ZBP1/necroptosis signaling in macrophages. These findings may help us explain the role of PTRF in allergic inflammation in new perspectives.

A recent study demonstrates that amino-terminal p40 fragment GSDMD, whose generation was independent of inflammatory caspase-1 and caspase-11, dominates cytosolic secretion of IL-33 by forming pores in the cell membrane in A549 and MLE-12 cells (lung epithelial type II cells) [1]. In our study, we did not find that HDM could induce cleavage of GSDMD and pro-IL-1β in bronchial epithelial cells, which is inconsistent with the study reported by Ge et al. [23]. Whether HDM induces p40 GSDMD fragmentation in bronchial epithelial cells warrants further investigation.

This study has several limitations. First, we conducted experiments using *Cavin-1*^*+/*–^ mice since *Cavin-1*^*–/–*^ mice exhibit a very low birth rate and growth problems. Second, the location of PTRF expression was unknown in our study. How did HDM regulate PTRF expression? How did PTRF regulate *Zbp1* expression? These questions will be investigated in our future research. The deletion of *Cavin-1* in macrophages might help us understand the role PTRF in mediating airway inflammation.

Taken together, PTRF positively regulates HDM-induced IL-33 expression in bronchial epithelial cells. Concurrently, PTRF increases ZBP1 expression and necroptosis signaling in HDM-challenged macrophages. Furthermore, bronchial epithelial cell-derived IL-33 works synergistically with HDM to enhance ZBP1 expression and necroptosis in HDM-challenged macrophages. Therefore, we conclude that PTRF-IL33-ZBP1 signaling mediating macrophage necroptosis contributes to HDM-induced airway inflammation. Our findings highlight the critical role of PTRF via regulating ZBP1/necroptosis in macrophages to drive HDM-induced airway inflammation.

## Materials and methods

### Animals and Asthma model

The *Cavin-1*^*+/*–^ mice (B6.129S6-Ptrftm1Pfp/J) were kindly provided by Professor K. Liao from the Shanghai Institute of Biochemistry and Cell Biology [24]. Heterozygous mice (*Cavin-1*^*+/–*^) were crossed to breed *Cavin-1*^*+/+*^ and heterozygous mice. Since *Cavin-1*^*–/–*^ mouse had a very low birth rate and growth problems and *Cavin-1*^*+/*–^ mouse had a very low protein level of PTRF in the lung, we used *Cavin-1*^*+/*–^ mice to perform the experiments [8]. *Il33*^–/–^ mice were provided by F. Zheng from the Huazhong University of Science and Technology [25]. Mice were housed under specific-pathogen-free conditions for 12 h dark/light cycles. Mice had access to food and water *ad libitum*. All animal experiments were conducted in accordance with the Institutional Animal Care and Use Committee guidelines of the Institute Pasteur of Shanghai, Chinese Academy of Sciences (Animal Ethics Review Number: A2018052). Female WT*, Cavin-1*^*+/–*^, and *Il33*^–/–^ (6–8 wk old) mice were used for experiments. Females were randomly allocated to experimental groups and no blinding method was used for treatment. There was no animal exclusion criteria. The HDM-induced mouse asthma model was established, as described previously [26]. Mice were challenged with intranasal administrations of 35 μl [0.7 mg/ml phosphate-buffered saline (PBS)] of whole HDM protein extract (Greer Laboratory, Boston, Mass) for five consecutive days per week (days 1–5) in two weeks. Control animals received only PBS. The anesthesia was induced with an intraperitoneal (i.p.) injection of pentobarbital sodium (50 mg/kg) before the mice were sacrificed. Mice were euthanatized for analysis 24 h after the last HDM treatment. All experiments were repeated at least three times with similar sample sizes.

### Isolation and culture of bone marrow-derived macrophages

Primary BMDMs were isolated and cultured as described previously [27]. Briefly, mice were sacrificed using standard CO_2_ asphyxiation guidelines followed by cervical dislocation. Using an aseptic technique, bone marrow was harvested from the femur and tibia bones. The marrow cavities were flushed with RPMI 1640 medium and bone marrow was collected. After centrifuging the blood samples at 300 *g* for 5 min and eliminating erythrocytes, the remaining cells were resuspended in a complete macrophage culture medium (CMCM, RPMI 1640 containing 10% FBS, 20% L929 cell-conditioned medium, 100 IU/ml penicillin and 100 μg/ml streptomycin). Cells were seeded at 37 °C with 5% CO_2_ for 7 days and CMCM was replaced on days 3 and 5. BMDMs were collected for the experiment on day 7 of culture.

### Cell culture and transfection

All cell lines were cultured in a humidified incubator at 37 °C with 21% O_2_ and 5% CO_2_. Raw 264.7, BEAS-2B, L929, and HEK293T cells were purchased from ATCC (American Type Culture Collection Manassas, USA) and grown in Dulbecco’s modified eagle medium (DMEM) supplemented with 10% fetal bovine serum (FBS), 2 μM glutamine, 100 μg/ml streptomycin sulfate, and 100 IU/ml penicillin. HUVEC were purchased from ScienCell and cultured in an endothelial cell growth medium (ScienCell, USA) containing 100× endothelial cell growth supplement (ScienCell, USA) and 5% FBS. All cell lines were recently authenticated by STR profiling, and the mycoplasma contamination test of all cells was negative. HDM protein extract (Greer Laboratory, Boston, Mass) and recombinant Mouse IL-33 (rmIL-33, Biolegend, 580506) were used for stimulating Raw264.7 or BMDMs.

For the gene knockdown, short hairpin RNA (shRNA) sequences, synthesized by Nanjing Genesis Biotechnology, were inserted into pLKO.1 plasmid between the EcoRI and NheI restriction sites. The shRNA PLKO.1 construct was introduced into target cells via lentiviral transduction. The knockdown assay primers were Scrambled 5-CAACAAGATGAAGAGCACCAA; mouse *Cavin-1* AGGTCAGCGTCAACGTGAAGA; human *CAVIN-1* GTGGAGGTTGAGGAGGTTATT; mouse *Zbp1* CCTGTATTCCATGAGAAATAA.

### Airway resistance index

AHR was measured using the Lung Function System (AniRes2005 V3.5, Animal Pulmonary Function Analysis System, Beijing Bestlab High-Tech Co., Ltd, China) 24 h after the last treatment. Briefly, the mice were anesthetized and connected to a pressure transducer via a tracheal cannula. Increasing concentrations of methacholine (0.025, 0.05, 0.1, and 0.2 mg/kg body weight) were injected into the external jugular vein at 5-min intervals using a fine needle. Resistance of the lung (RL), resistance to expiration (Re), and dynamic respiratory compliance (Cdyn) were recorded to evaluate the airway reactivity.

### Bronchoalveolar lavage fluid collection

Lavage of the lungs was performed by flushing with 1 ml PBS 3 times at the end of experiments. After centrifuging, the supernatant was used to measure LDH with LDH Cytotoxicity Assay Kit and total protein concentration by Pierce BCA assay (Thermo Scientific, Waltham, MA, USA) according to the manufacturer’s instructions. The cell pellet from BALF was then resuspended in PBS and analyzed for total cell count with TC20 automated cell counter (Bio-Rad Inc, Hercules, California). The remaining BALF cells were stained for Flow cytometric analysis.

### Isolation of mouse lung cells

After anesthetization, mice were performed tracheal intubation, and then blood was taken by exsanguinating mice from the Vena Cava. Blood plasma was performed for IgE measurement. After BALF was collected, 1 ml of dispase II (2 U/ml) was injected through the trachea [28]. Subsequently, the lungs were incubated and digested in 2 ml of 2 μg/ml collagenase/dispase II containing 0.001% DNAse 1 for 30 min at 37 °C on a shaker. Cells were collected by centrifugation at 335 × *g* for 10 min at 4 °C in a 15 ml conical tube. ACK lysis buffer was used for lysing erythrocytes, and the cells were rinsed twice with cold PBS/0.5% BSA. After being resuspended in 1 ml cold PBS/0.5% BSA, single cells were passed through a 70 μm cell strainer and collected for flow cytometry. Total cells were diluted and counted by the TC20 automated cell counter*.*

### Flow cytometry

The methods of flow cytometry adhere to the guidelines [29]. BALF cells and lung cells were prepared as described previously. For cell surface staining, cell suspensions were incubated with the antibody cocktails for 30 min at 4 °C. For intracellular cytokine staining, cells were stimulated with Leukocyte Activation Cocktail for 4 h (BD Pharmingen, 550583), which contained 50 ng/ml phorbol12-myristate 13-acetate (PMA), 1 μg/ml ionomycin and 1 μg/ml GolgiPlugTM protein transport inhibitor. Then, Cytofix/Cytoperm Kit (BD Pharmingen, 554714) was used to intracellular cytokines staining. For nuclear protein staining, cells were treated with Transcription Factor Buffer Set (BD Pharmingen, 562574) according to the manufacturer’s instructions. Purified rat anti-mouse CD16/CD32, anti-FVS-BV570, anti-CD45.1-APC-Cy7, anti-CD11b-PerCP-Cy5.5, anti-F4/80-PE, anti-Ly6C-BV711, anti-Ly6G-PE-Cy7, anti-CD3-FITC, anti-CD4-PerCP, anti-GATA3 -BV421, anti-IL-17A-APC antibodies and mouse anti-Siglec-F-BV421 antibody were used. Flow cytometry experiments were acquired on LSRFortessa^TM^ Flow

Cytometer and all data was analyzed in FlowJo (Tree Star, Ashland, OR). The gating strategies for each cell population are listed in the Supplementary Figs. 4–5.

### Quantitative real-time PCR

Total RNA was extracted from lung homogenates or cultured cells using TRIzol reagent (Invitrogen, Carlsbad, CA) following the manufacturer’s instructions. cDNA was generated using a reverse transcriptase kit (FastQuant RT Kit, Tiangen Biotech), followed by quantitative RT-PCR analysis (SYBR Green, TaKaRa). The plate was analyzed on the ABI 7900HT Fast Real-Time PCR System using a 10 μl reaction volume. The relative expression levels of target genes were determined by the 2^-△△Ct^ cycle threshold method. The primers of *Il4, Il5, Il13, Il17a, Gob5, Muc5ac, Zbp1, Ripk1, Ripk3, Mlkl, Tnfα, Il6, Il1b, Cavin-1, Il33*, and *Gapdh* were listed as follows:

**Table:** 

Mouse Gene	Forward	Reverse
*Il4*	TTGAGAGAGATCATCGGCATTTTG	TCAAGCATGGAGTTTTCCCATGT
*Il5*	TGTTGACAAGCAATGAGACGATGA	AATAGCATTTCCACAGTACCCCCA
*Il13*	CGGCAGCATGGTATGGAGTGTG	GGAGGCTGGAGACCGTAGTGG
*Il17a*	CTCCAGAAGGCCCTCAGACTAC	AGCTTTCCCTCCGCATTGACACAG
*Gob5*	ACTAAGGTGGCCTACCTCCAA	GGAGGTGACAGTCAAGGTGAGA
*Muc5ac*	CCATGCAGAGTCCTCAGAACAA	TTACTGGAAAGGCCCAAGC
*Zbp1*	AAGAGTCCCCTGCGATTATTTG	TCTGGATGGCGTTTGAATTGG
*Ripk1*	GACAGACCTAGACAGCGGAG	CCAGTAGCTTCACCACTCGAC
*Ripk3*	TCCCAATCTGCACTTCAGAAC	GACACGGCACTCCTTGGTAT
*Mlkl*	AGGAACCAGTGGGTCAGGAT	CAAGATTCCGTCCACAGAGGG
*Tnfα*	CCCACGTCGTAGCAAACCAC	GCAGCCTTGTCCCTTGAAGA
*Il6*	GGCCTTCCCTACTTCACAAG	ATTTCCACGATTTCCCAGAG
*Il1b*	CACAGCAGCACATCAACAAG	GTGCTCATGTCCTCATCCTG
*Cavin-1*	AGTGAGCTCAAAGCCAGCAT	GCCTTAGTTCCCCCAAAGAC
*Il33*	CTGGCCTCACCATAAGAAAGGAGA	AGGGAGGCAGGAGACTGTGTTAAA
*GAPDH*	CCCACTAACATCAAATGGGG	CCTTCCACAATGCCAAAGTT
Human Gene	**Forward**	**Reverse**
*CAVIN-1*	GAGGACCCCACGCTCTATATT	CCCCGATGATTTTGTCCAGGA
*GAPDH*	GGAGCGAGATCCCTCCAAAAT	GGCTGTTGTCATACTTCTCATGG

### Western blotting

The lung homogenates and cells were lysed in RIPA buffer (Beyotime) containing protease inhibitor (Bimake). Total protein was measured with Pierce BCA assay and equal amounts of denatured proteins were loaded and run on 10 to 12.5% gradient gel. After transferred to PVDF membranes, the protein was hybridized with indicated primary antibodies and corresponding HRP labeled secondary antibodies. Super ECL Substrate (Tanon, Shanghai, China) was used to detect the bands, which were analyzed and quantitated using Image J software. The primary antibody used in western blot analysis were as follows: Anti-tubulin (11224-1-AP; proteintech); anti-GAPDH (10494-1-AP; proteintech); anti-PTRF (ab48824; Abcam); anti-ZBP1 (sc-271438; Santa Cruz Biotechnology); anti-RIP3 (phospho T231 + S232) (2D7, ab205421; Abcam); anti-RIP3 (sc-374639; Santa Cruz Biotechnology); anti-mouse MLKL (phospho S345) (EPR9515(2), ab196436; Abcam); anti-human MLKL (phospho S358) (EPR9514, ab1187091; Abcam); anti-MLKL (ab243142; Abcam); anti-IL-1β (ab9722; Abcam); anti-caspase-3 (9662 S; CST); anti-GSDMD (ab209845; Abcam); anti-mouse IL-33 (AF3626; R&D Systems); anti-human IL-33 (ab54385; Abcam). The original Western blotting gel images are listed in the Supplementary Materials.

### Enzyme-linked immunosorbent assay and LDH release assay

Whole lungs were homogenized in 1 ml PBS containing 0.05% Triton X-100, Pierce Protease and Phosphatase Inhibitor cocktail (Thermo Scientific™). Suspensions were filtered with a 40 μm cell strainer and clarified by centrifugation. Supernatant was used for detecting cytokines by ELISA [30]. The levels of IL-4, IL-5, IL-13, IL-33, and IgE were measured with sandwich ELISA kit according to the manufacturer’s instructions. For IL-33 in lung homogenate, also total protein determination was performed from the same sample, and the results were then presented in relation to total protein concentration for each sample. LDH release was detected in BALF and cells supernatant using the LDH Cytotoxicity Assay Kit (88953; Thermo Scientific) according to the manufacturer’s instructions. Readings were carried out at a 490 nm wavelength, using a microplate reader (Thermo Scientific) and expressed as % LDH release.

### Propidium iodide staining

Cells were cultured in 12-well plates, stimulated with PBS or HDM for 24 h, washed twice with cold PBS, and then levitated in 1 mL binding buffer. To observe the cell membrane integrity, the cells were incubated with PI (5 ng/mL) at room temperature for 15 min avoiding light. The fluorescence signals were analyzed by flow cytometer immediately. Static bright-field photos of dead cells were captured and processed by Olympus IX73 and Image J software (Tree Star, Ashland, OR). Quantification of mean fluorescence intensity of PI staining cells using PBS-treated cells with staining as a background.

### Lung histology

Mouse lung tissues were fixed in 4% formaldehyde overnight at RT. After embedded in paraffin, tissues were cut into 5 μm sections and used for hematoxylin and eosin (HE) and periodic acid-schiff (PAS) staining. Images were taken using CaseViewer software version 3 (DHISTECH, Hungary). The lung inflammation score was determined in a blinded manner, as described previously [31].

### RNA-Seq and RNA-Seq data analysis

The wildtype, *Cavin-1*^–/–^, and *Il33*^–/–^ mice were treated with either PBS or HDM. At designed timepoints, the mice were sacrificed. The lungs were removed and homogenized to extract the total RNA in TRIzol reagent (Invitrogen, Carlsbad, CA). After conducted quantification and qualification, the total RNA of each sample was sequenced using the Illumia platform (Novogene Co., LTD). STRING Database (https://string-db.org) was assessed to analyze the targeted genes which were differentially expressed in our study. GraphPad Prism 8.0.1 (San Diego, CA) was used to analyze difference of genes by Volcano plot.

### Statistical analysis

We conducted statistical analysis with GraphPad Prism version 6.0 software (GraphPad, San Diego, CA, USA). The results were presented as means ± standard error of the mean (SEM). *P* values were calculated with student’s t tests (independent two-sample) and one-way analysis of variance (ANOVA) with the Tukey’s correction for post hoc paired comparisons (independent samples more than three). *P* value < 0.05 was considered statistically significant.

## Supplementary information

supplemental materials

original data files

aj-checklist

## Data Availability

All data that support the findings of this study are available from the corresponding author upon request.
