# Supplementary material for: PTRF-IL33-ZBP1 signaling mediating macrophage necroptosis contributes to HDM-induced airway inflammation
Source: Cell Death Dis. 2023 Jul 15;14(7):432. doi: 10.1038/s41419-023-05971-1 (PMC10349813; doi:10.1038/s41419-023-05971-1)
Supplement: Supplementary file 1 — supplemental materials [file 41419_2023_5971_MOESM1_ESM.docx]

**Supplementary Materials**

**PTRF-IL33-ZBP1 signaling mediating macrophage necroptosis contributes to HDM-induced airway inflammation**

Juan Du^#1, 2^, Yahui Liu^#1, 2^, Gelei Lan^1, 2^, Yao Zhou^3^, Yingmeng Ni^1, 2^, Kan Liao^4^, Fang Zheng^5^, Qijian Cheng^1, 2^, Guochao Shi^1, 2*^, Xiao Su^6,7*^

**Supplementary Figure 1**

**Mouse genotyping and confirmation by RT-PCR and Western blotting.** (A) Identification of mouse genotypes with PCR. (B) *Cavin-1* mRNAs in lung was assessed by RT-qPCR (normalized to GAPDH). (C) Protein level of PTRF in lung was assessed by Western blot. Data are means ± SD, ***P* < 0.01, ****P* < 0.001 as calculated by two-tailed unpaired student’s t-test. n=3 mice in each group.

**Supplementary Figure 2**

**Western analysis of ZBP1/necroptosis signaling pathway and IL-33 expression in HDM-challenged BEAS-2B** **and HUVEC cells.** **A-B.** **Western analysis of ZBP1/necroptosis signaling pathway and IL-33 expression in BEAS-2B**. (A) The BEAS-2B cells were stimulated with indicated concentration of HDM for 24 h. Western blot analysis of PTRF, ZBP1 and necroptosis markers p-RIPK3 and p-MLKL in cells lysates. (B) BEAS-2B cells were transfected with scrambled shRNA or *CAVIN-1* shRNA1 and stimulated with 50 μg/ml HDM for 24 h. Western blot analysis of PTRF, ZBP1 and necroptosis markers p-RIPK3 and p-MLKL, and IL-33 in cell lysates. Data were from three independent experiments. **C-D. Western analysis of ZBP1/necroptosis signaling pathway in HUVEC**. (A) The HUVEC cells were stimulated with indicated concentration of HDM for 24 h. Western blot analysis of PTRF, ZBP1 and necroptosis markers p-RIPK3 and p-MLKL in cells lysates. (B) HUVEC cells were transfected with scrambled shRNA or *CAVIN-1* shRNA1 and stimulated with 50 μg/ml HDM for 24 h. Western blot analysis of PTRF, ZBP1 and necroptosis markers p-RIPK3 and p-MLKL in cell lysates.

**Supplementary Figure 3**

**Deletion of *Il33* lessens HDM-induced airway inflammation.** The mice were sacrificed at endpoint of experiments. (A) Total cell counts and protein levels in BALF, and plasma IgE levels were measured by ELISA. (B) Histological examination of mouse paraffin lung sections stained with H&E (upper panel) and PAS (lower panel). Scale bar, 100μm. (C) Inflammation scores of lung tissues and analysis of PAS-positive cells. (D) IL-4, IL-5 and IL-13 in lung homogenates were measured by ELISA. Data are means ± SD, n=4-6 mice in each group. **P* < 0.05, ***P* < 0.01, ****P* < 0.001, *****P* < 0.0001 as calculated by two-tailed unpaired student’s t-test, corrected by one-way ANOVA with Turkey post-hoc test.

**Supplementary Figure 4**

**Exemplary gating strategy to identify cells by flow cytometry.** (A) Eosinophils in BALF. (B) Neutrophils in BALF. (C) Eosinophils in lungs. (D) Macrophages in lungs. (E) Th2 cells in lungs. (F) Th17 cells in lungs.

**Supplementary Figure 5**

**Gating strategy to identify PI^+^ cells by flow cytometry**

**Supplementary Figure 6**

**Positive control for presenting antibodies**
