# Supplementary material for: PTRF-IL33-ZBP1 signaling mediating macrophage necroptosis contributes to HDM-induced airway inflammation
Source: Cell Death Dis. 2023 Jul 15;14(7):432. doi: 10.1038/s41419-023-05971-1 (PMC10349813; doi:10.1038/s41419-023-05971-1)

Supporting Figure_3

G

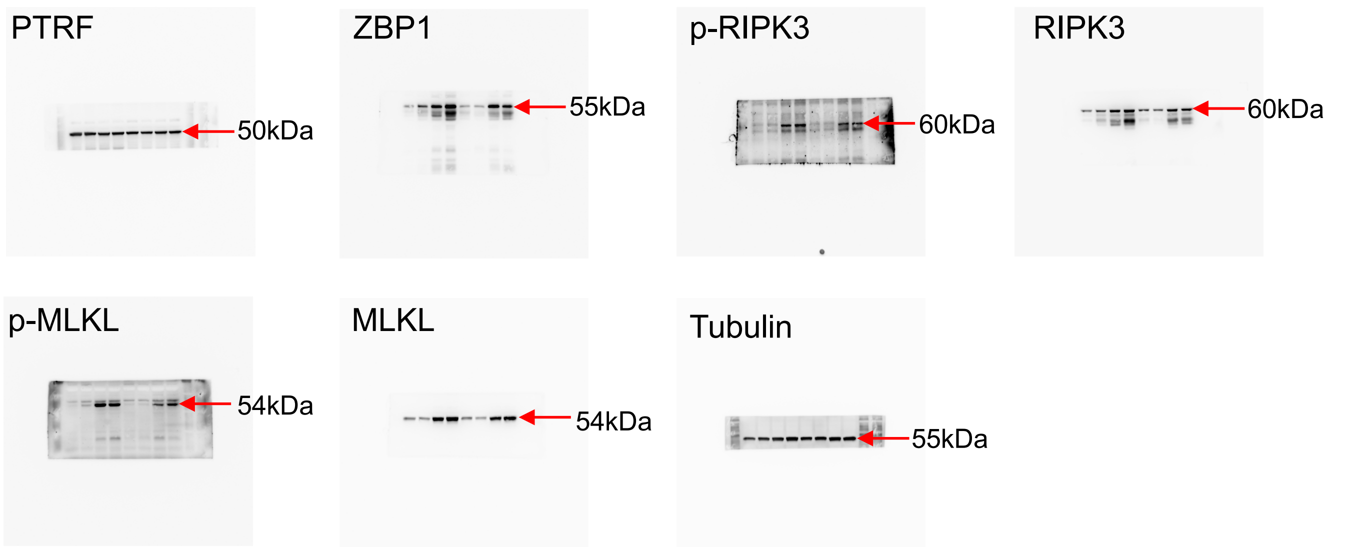

H

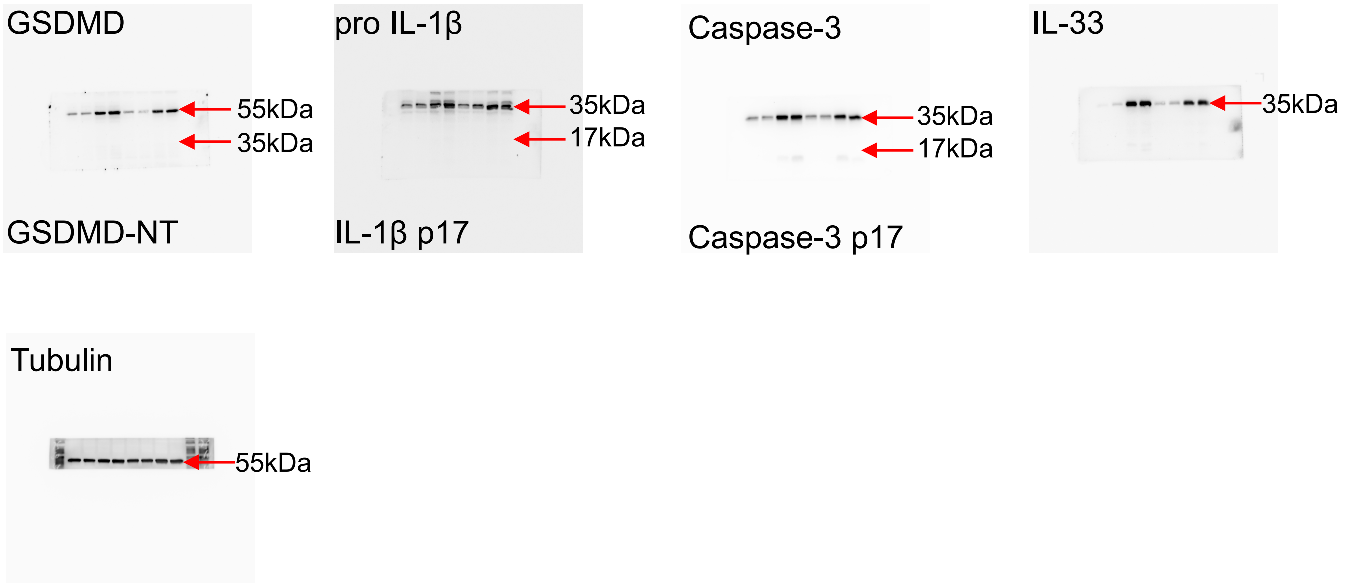

Supporting Figure_4

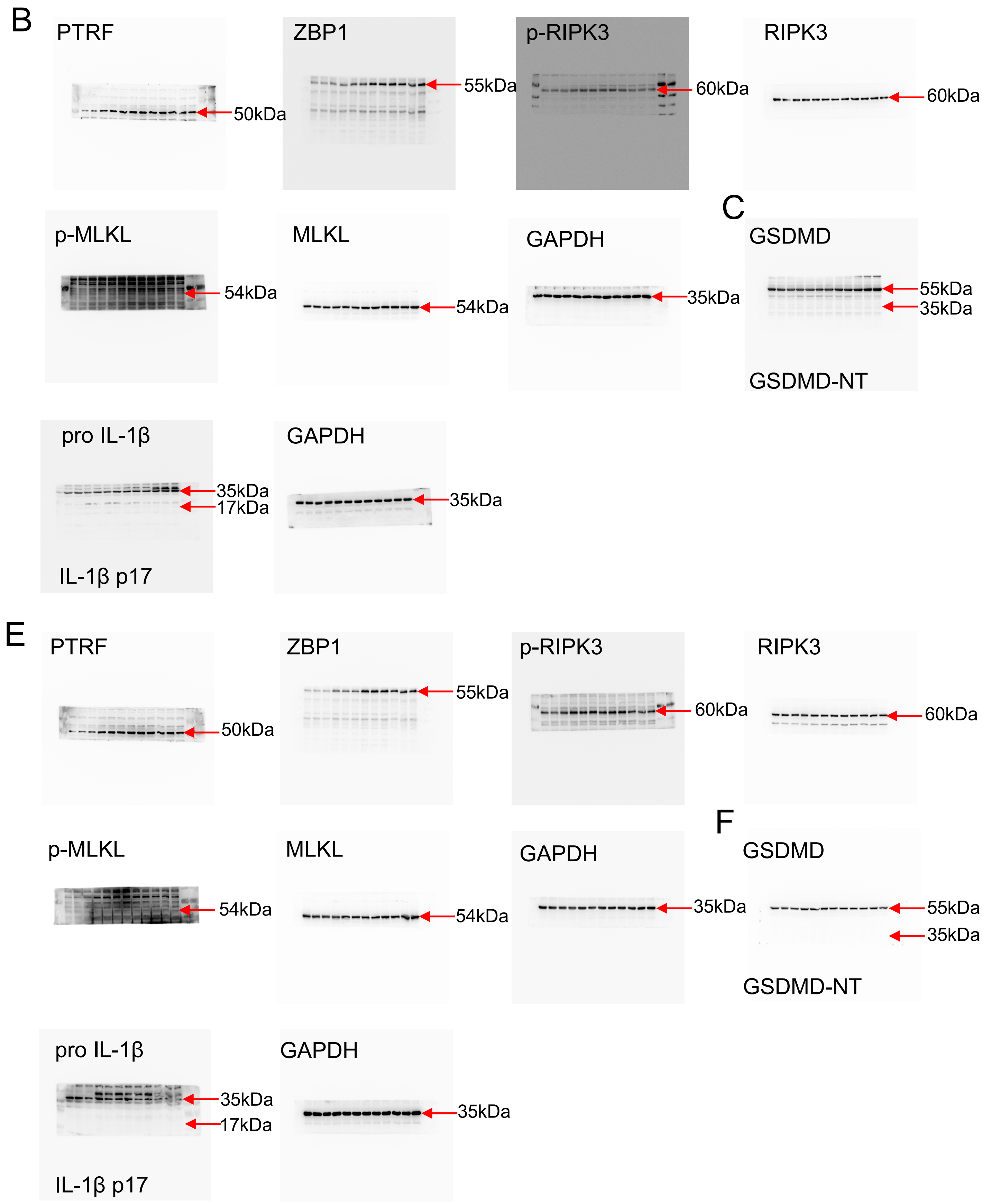

Supporting Figure_5

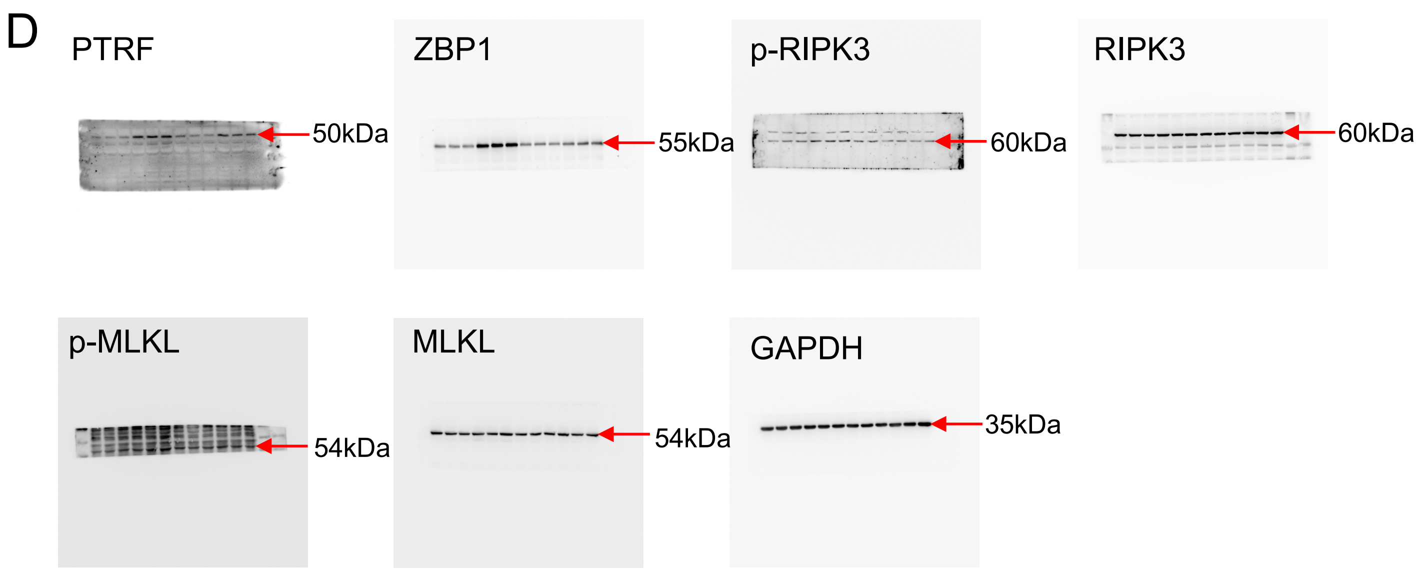

Supporting Figure_6

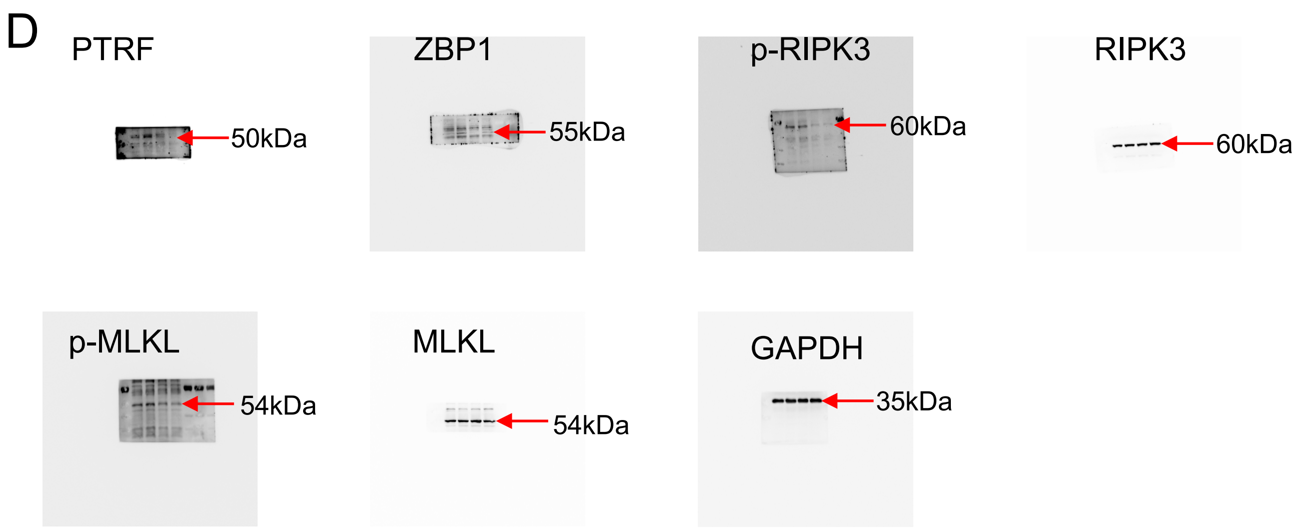

Supporting Figure_7

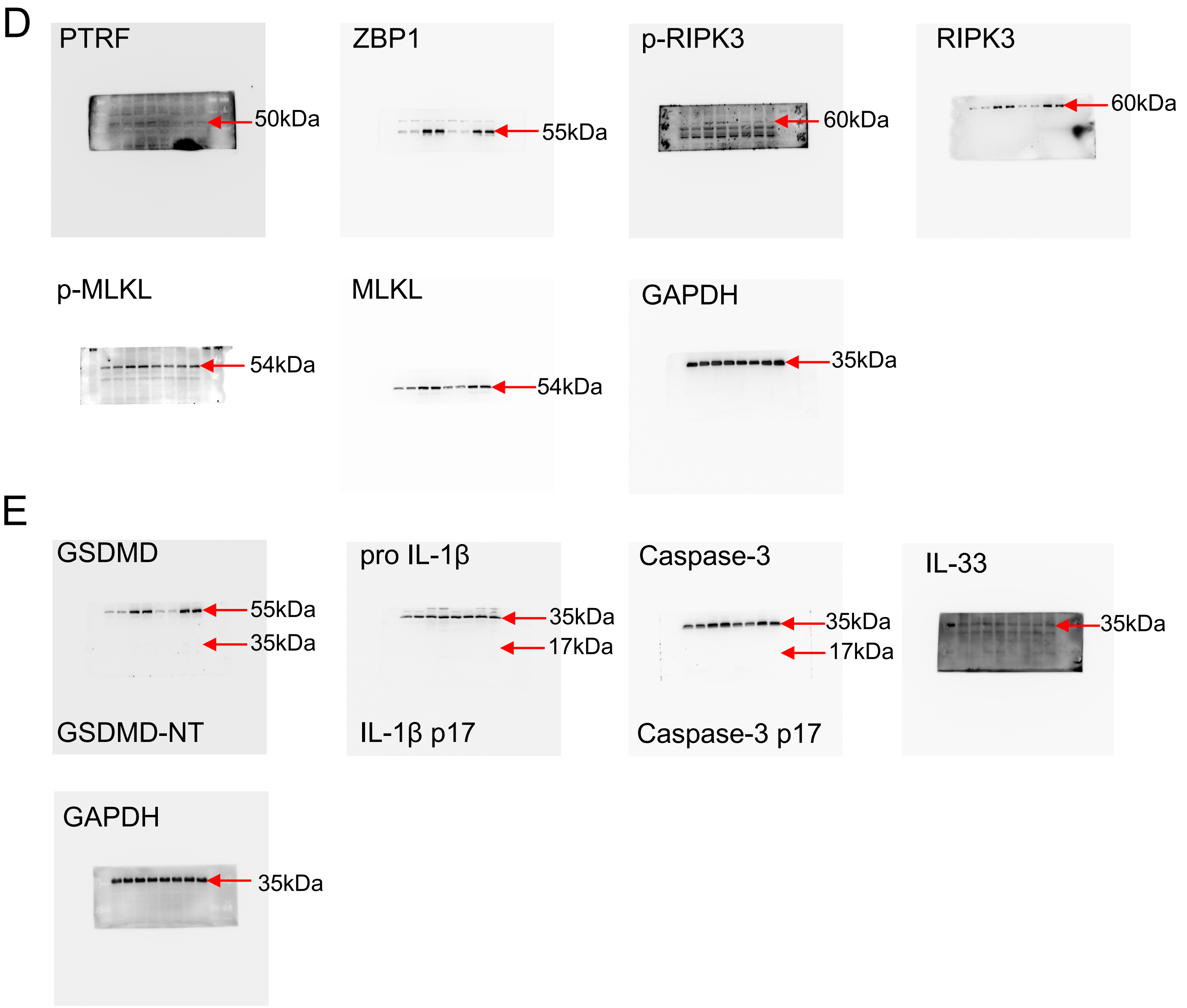

Supporting Figure_8

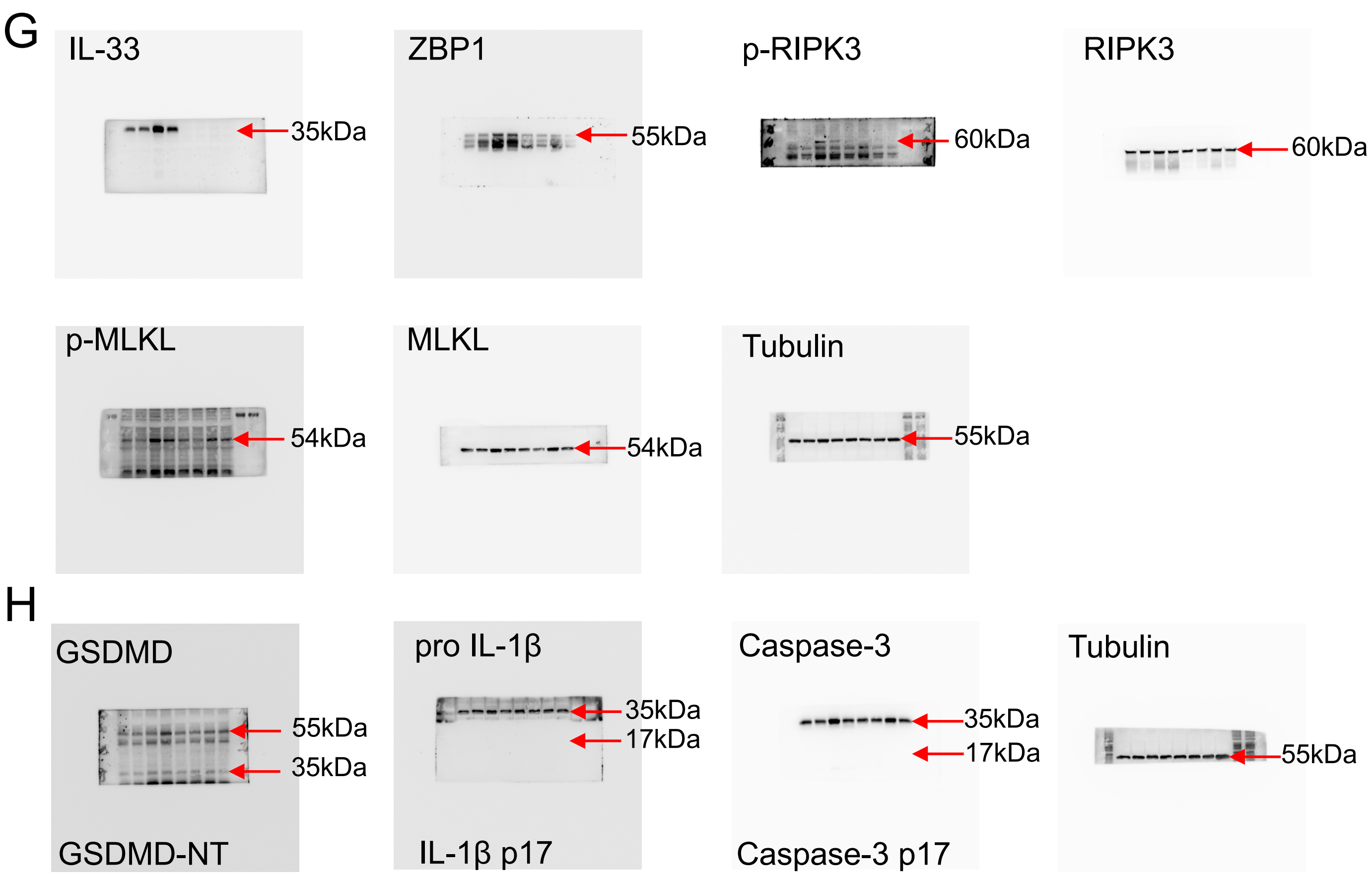

Supporting Figure_9

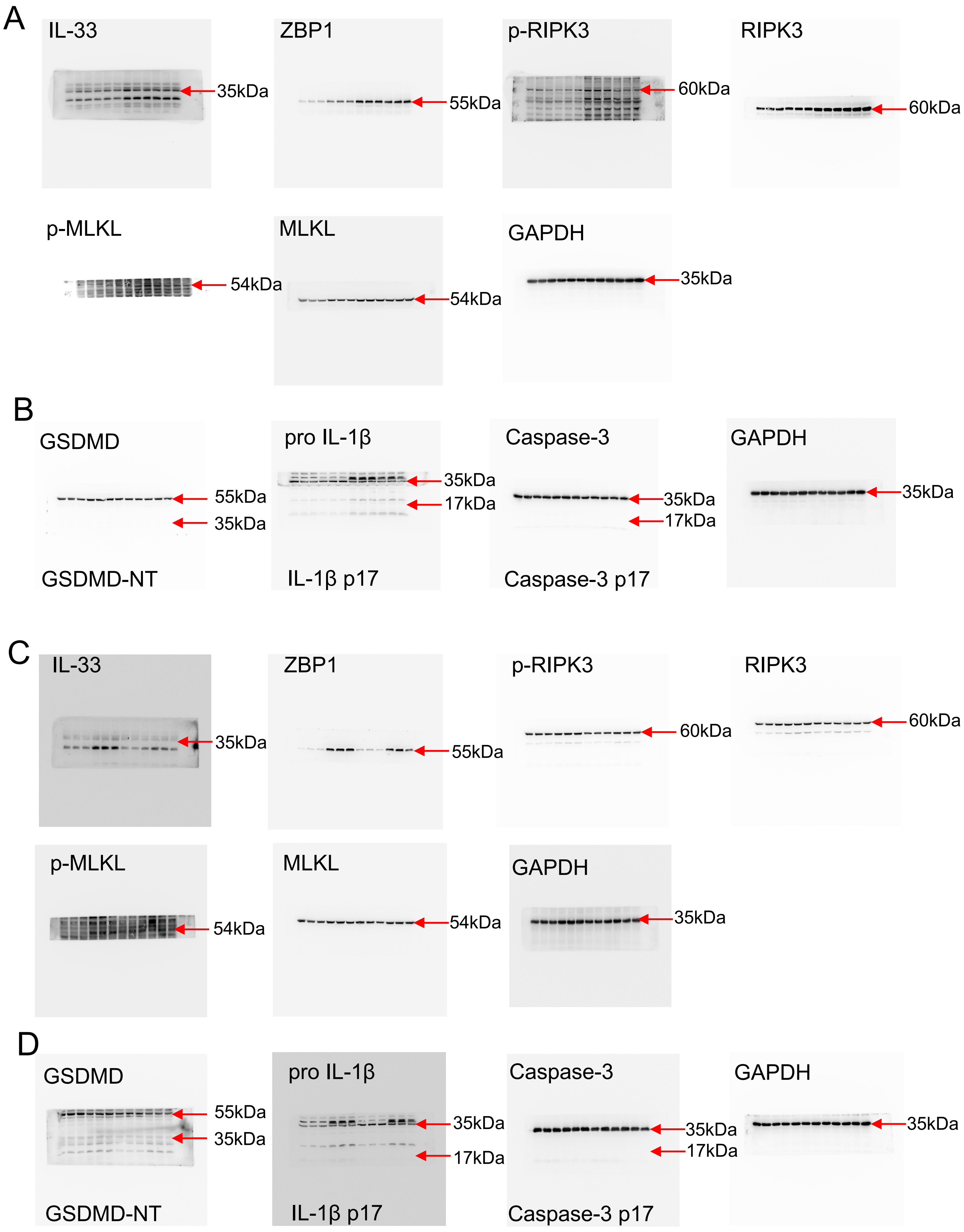

Supplement: Supplementary file 2 — original data files [file 41419_2023_5971_MOESM2_ESM.pdf]
